# Neuroprotection against ischemic stroke requires a specific class of early responder T cells in mice

**DOI:** 10.1172/JCI157678

**Published:** 2022-08-01

**Authors:** Wei Cai, Ligen Shi, Jingyan Zhao, Fei Xu, Connor Dufort, Qing Ye, Tuo Yang, Xuejiao Dai, Junxuan Lyu, Chenghao Jin, Hongjian Pu, Fang Yu, Sulaiman Hassan, Zeyu Sun, Wenting Zhang, T. Kevin Hitchens, Yejie Shi, Angus W. Thomson, Rehana K. Leak, Xiaoming Hu, Jun Chen

**Affiliations:** 1 Pittsburgh Institute of Brain Disorders and Recovery, and Department of Neurology, School of Medicine, University of Pittsburgh, Pittsburgh, Pennsylvania, USA.; 2 Geriatric Research, Education and Clinical Center, Veterans Affairs Pittsburgh Health Care System, Pittsburgh, Pennsylvania, USA.; 3 Animal Imaging Center and Department of Neurobiology, School of Medicine, University of Pittsburgh, Pittsburgh, Pennsylvania, USA.; 4 Starzl Transplantation Institute, Department of Surgery, and Department of Immunology, University of Pittsburgh School of Medicine, Pittsburgh, Pennsylvania, USA.; 5 Graduate School of Pharmaceutical Sciences, Duquesne University, Pittsburgh, Pennsylvania, USA.

**Keywords:** Inflammation, Neuroscience, Immunotherapy, Stroke, T cells

## Abstract

Immunomodulation holds therapeutic promise against brain injuries, but leveraging this approach requires a precise understanding of mechanisms. We report that CD8^+^CD122^+^CD49d^lo^ T regulatory-like cells (CD8^+^ TRLs) are among the earliest lymphocytes to infiltrate mouse brains after ischemic stroke and temper inflammation; they also confer neuroprotection. TRL depletion worsened stroke outcomes, an effect reversed by CD8^+^ TRL reconstitution. The CXCR3/CXCL10 axis served as the brain-homing mechanism for CD8^+^ TRLs. Upon brain entry, CD8^+^ TRLs were reprogrammed to upregulate leukemia inhibitory factor (LIF) receptor, epidermal growth factor–like transforming growth factor (ETGF), and interleukin 10 (IL-10). LIF/LIF receptor interactions induced ETGF and IL-10 production in CD8^+^ TRLs. While IL-10 induction was important for the antiinflammatory effects of CD8^+^ TRLs, ETGF provided direct neuroprotection. Poststroke intravenous transfer of CD8^+^ TRLs reduced infarction, promoting long-term neurological recovery in young males or aged mice of both sexes. Thus, these unique CD8^+^ TRLs serve as early responders to rally defenses against stroke, offering fresh perspectives for clinical translation.

## Graphical abstract

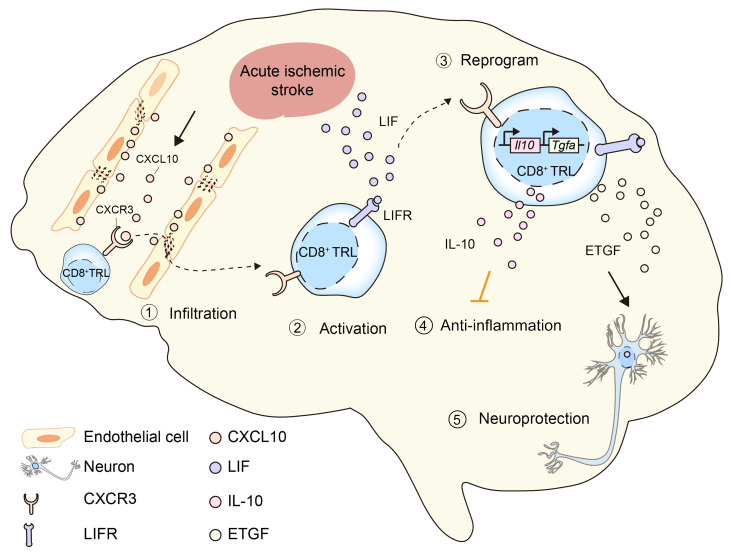

## Introduction

Immune responses play diverse roles in brain injury and repair after stroke. The sudden occlusion of a cerebral vessel leads to acute ischemic damage, followed by immediate activation of local immune cells and prompt mobilization of peripheral immune cells (1). Initially, innate immune cells function to restrict brain damage by clearing cell debris and neutralizing neurotoxins. However, detrimental products released from overactivated immune cells can lead to injury augmentation (1). Thus, there is substantial new interest in therapeutic approaches that harness the power of immune responses. In particular, regulatory lymphocyte subpopulations that infiltrate the ischemic brain, including CD4^+^CD25^+^ regulatory T cells (CD4^+^ Tregs) (2, 3) and interleukin 10–positive (IL-10^+^) regulatory B cells (Bregs) (4), may modulate endogenous protective responses to injury and are therefore being explored as therapeutic approaches for stroke treatment. For example, CD4^+^ Tregs protect against ischemic brain injury and promote brain repair after stroke (3–6). Another T lymphocyte population, the CD8^+^CD122^+^ T cell, is critical in regulating immune responses in autoimmune and inflammatory diseases, including experimental autoimmune encephalomyelitis and colitis (7, 8). Although acute brain injury sets many inflammatory cascades in motion, the function of CD8^+^CD122^+^ T cells in ischemic stroke is uncharted territory.

CD8^+^CD122^+^ T cells were originally recognized as antigen-specific memory T cells. They are divided into effector memory T cells and central memory T cells, based on differential expression of surface markers such as CD62L, CD44, and CCR7 (9). Emerging evidence shows that a specialized population of CD8^+^CD122^+^ T cells perform regulatory functions (9). These regulatory CD8^+^CD122^+^ T cells are distinguished from memory CD8^+^CD122^+^ T cells by low expression of CD49d (10) and high expression of programmed death-1 (11). The primary function of regulatory CD8^+^CD122^+^ T cells is to suppress proliferation and proinflammatory effects of other immune cells, especially effector T (Teff) lymphocytes, and to maintain immune homeostasis. Usually, direct interactions between regulatory CD8^+^CD122^+^ T cells and their target cells are sufficient to induce regulatory action, and no interaction with antigen-presenting cells is required. CD8^+^CD122^+^ T cells may exert regulatory functions by releasing protective factors such as IL-10 and/or through direct cell-cell contact (12). Therefore, these cells may provide fast, non–antigen-specific immunoregulation (13). Compared with their CD4^+^CD25^+^ Treg counterparts, regulatory CD8^+^CD122^+^ T cells exhibit more potent immunosuppressive activity (14). In one study, the neuroprotective effect of IL-10^+^ Bregs in a stroke model was accompanied by increased CD8^+^CD122^+^ T cells in the brain and spleen (15), suggesting a possible regulatory role of this cell population in the ischemic brain.

The current study reports what we believe is a new subpopulation of CD8^+^CD122^+^ T cells that we define here as “T regulatory-like cells” (CD8^+^ TRLs). CD8^+^ TRLs enter the ischemic brain early after stroke, preceding the infiltration by CD4^+^ Tregs. Selective depletion of circulating CD8^+^CD122^+^ T cells doubled the ischemic infarct volume and amplified sensorimotor deficits by 2- to 3-fold in a mouse model of stroke. We report CXCR3 as an important receptor mediating early recruitment of CD8^+^ TRLs into the ischemic brain. These cells are different from previously described CD8^+^CD122^+^ Tregs in that they adopt a distinct neuroprotective effector state upon brain infiltration and release protective/immunomodulatory factors in brain tissues, including epidermal growth factor–like transforming growth factor (ETGF) and IL-10 in a leukemia inhibitory factor receptor–dependent (LIFR-dependent) manner. CD8^+^ TRLs are uniquely versatile in that they serve as cellular sentinels of early tissue damage, as well as the gatekeepers of downstream protective responses.

## Results

### Early responder CD8^+^ TRLs infiltrate the ischemic brain within 1 day after stroke and linger in the injured brain for at least 2 weeks.

**Figure 1:**
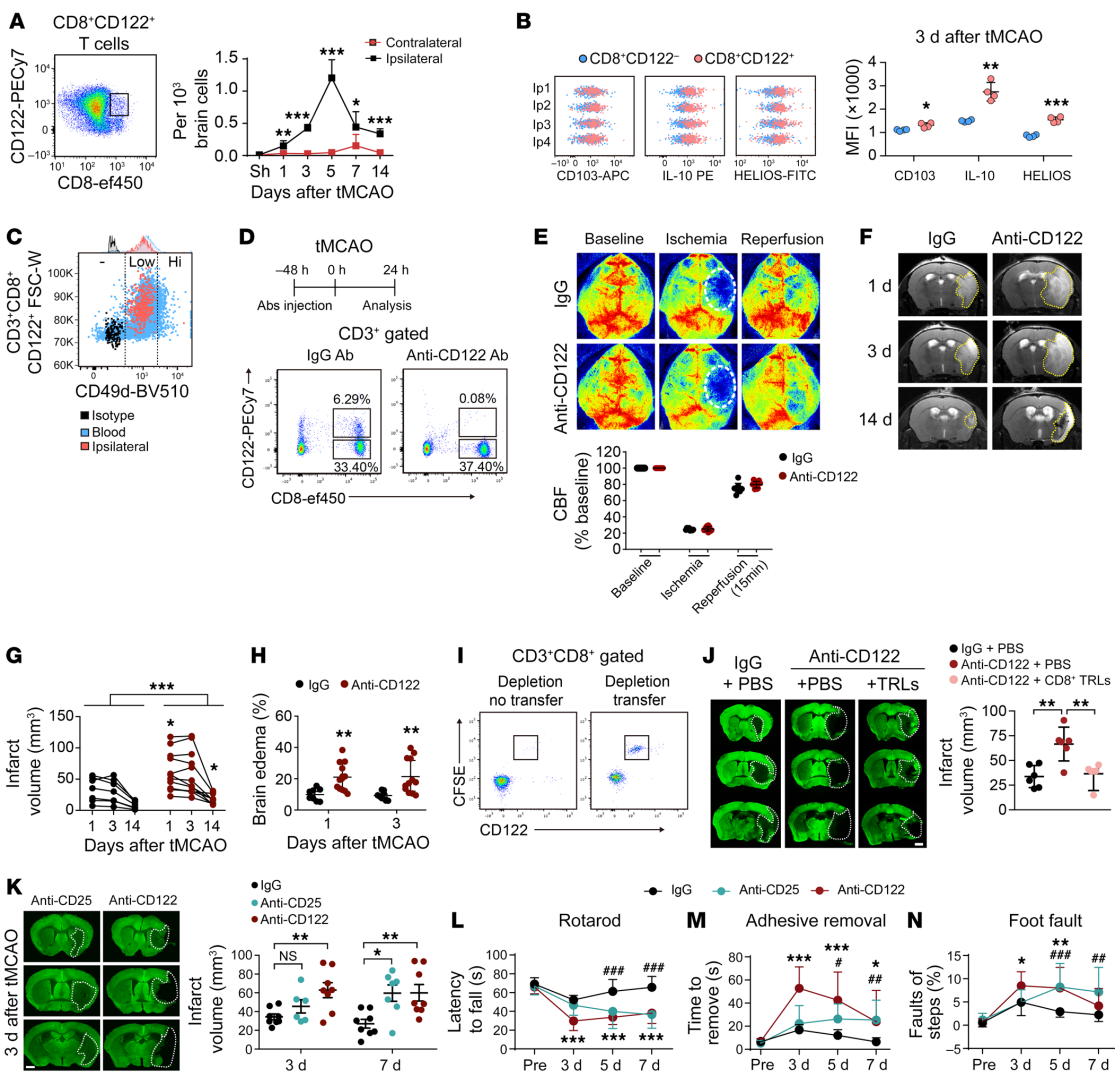
Early infiltration of CD8^+^ TRLs provides protection against cerebral ischemia. (**A**) CD8^+^CD122^+^ T cell infiltration in the ipsilateral and contralateral brains at indicated time points after 60-minute tMCAO. *n =* 4–7/group. Two-tailed Student’s *t* test. (**B**) Expression of CD103, IL-10, and HELIOS in brain-infiltrating CD8^+^CD122^+^ T cells and CD8^+^CD122^–^ T cells 3 days after tMCAO. *n =* 4/group. Two-tailed Student’s *t* test. (**C**) Most of the infiltrating CD8^+^CD122^+^ T cells are CD49d^lo^ TRLs. (**D**–**H**) Mice were treated with isotype IgG (100 μg) or anti-CD122 mAb (100 μg) 2 days prior to 60-minute tMCAO. *n =* 8–12/group. (**D**) Anti-CD122 mAb treatment depleted CD8^+^CD122^+^ T cells in blood 1 day after stroke. (**E**) Quantitative measurements of cortical CBF before MCAO (baseline), during MCAO, and 15 minutes after reperfusion. (**F**) Representative images of T2 MRI scans 1, 3, and 14 days after tMCAO. The yellow dotted lines depict the infarct areas. Quantification of brain infarcts (**G**) and brain edema (**H**). In **G**, black symbols indicate IgG treatment, red symbols indicate anti-CD122 treatment, and *P* = 0.06 for anti-CD122 versus IgG at 3 days after tMCAO. Two-way (**E** and **H**) or mixed-effects (**G**) repeated measures ANOVA and post hoc Bonferroni’s test. (**I**) FACS-isolated CD8^+^ TRLs were labeled with CFSE and adoptively transferred (1 million cells, i.v.) to recipient mice 48 hours after anti-CD122 mAb injection. CD8^+^CD122^+^CFSE^+^ cells were detected in blood 24 hours after cell transfer. (**J**) Adoptive transfer of CD8^+^ TRLs 2 hours after tMCAO reversed the detrimental effects of anti-CD122 mAb on brain infarction 3 days after tMCAO as shown by MAP2 staining. *n =* 6/group. One-way ANOVA and post hoc Dunnett’s test. Scale bar: 1 mm. (**K**) Quantification of infarct volumes 3 and 7 days after tMCAO in mice treated with IgG, anti-CD122 mAb, or anti-CD25 mAb (100 μg). *n =* 7–8/group. One-way ANOVA and post hoc Dunnett’s test. (**L**–**N**) Sensorimotor function was analyzed with the rotarod test (**L**), adhesive removal test (**M**), and foot-fault test (**N**). *n =* 8–10/group. Two-way repeated measures ANOVA and post hoc Dunnett’s test. *P < 0.05, **P < 0.01, ***P < 0.001 for anti-CD122 vs. IgG. ^#^*P <* 0.05, ^##^*P <* 0.01, ^###^*P <* 0.001 for anti-CD25 vs. IgG.

Inspired by recent findings of beneficial roles of regulatory lymphocytes in CNS diseases (2, 3), we investigated the temporal profiles of Treg cell infiltration of the ischemic mouse brain. The results show that significant infiltration by CD8^+^CD122^+^ T cells of the ischemic hemisphere began as early as 1 day after 60-minute transient middle cerebral artery occlusion (tMCAO) (Figure 1A and Supplemental Figure 1A; supplemental material available online with this article; https://doi.org/10.1172/JCI157678DS1), preceding the initial entry of CD4^+^ Tregs, which occurred 5 days after tMCAO (Supplemental Figure 1B). The number of CD8^+^CD122^+^ T cells peaked on day 5 and remained elevated in the ischemic brain until at least 14 days after stroke (Figure 1A). Immunostaining confirmed the presence of CD8^+^CD122^+^ T cells in the ischemic core and peri-infarct area 5 days after stroke (Supplemental Figure 1C). These cells expressed higher levels of HELIOS compared with CD8^+^CD122^–^ T cells (Figure 1B), which is required for the inhibitory activity of Treg cells (16). Expression of CD103 and IL-10 was also higher in CD8^+^CD122^+^ T cells (Figure 1B). Further characterization confirmed that these infiltrating CD8^+^CD122^+^ T cells were CD49d^lo^ T regulatory–like cells (CD8^+^ TRLs) rather than CD49d^hi^ memory T cells (Figure 1C). In the periphery, the numbers of CD8^+^CD122^+^ cells in blood and spleen dropped significantly at 1 day, remained low at 3 days, and then gradually returned to prestroke levels (Supplemental Figure 1, D and E). The numbers of CD4^+^ Tregs in blood and spleen were 3- to 4-fold lower than CD8^+^CD122^+^ T cells under normal conditions. The blood CD4^+^ Tregs showed a reduction similar to that of CD8^+^CD122^+^ T cells early after stroke, followed by gradual recovery (Supplemental Figure 1E).

### CD8^+^ TRLs provide early protection to the ischemic brain and improve functional outcomes.

To explore the functional role of CD8^+^ TRLs in stroke, an anti-CD122 monoclonal antibody (mAb) was used to selectively deplete CD8^+^CD122^+^ T cells in the circulation (17). Adult male mice were randomly assigned to receive a single dose of anti-CD122 mAb (100 μg) or equivalent quantity of isotype IgG (IgG) intraperitoneally 2 days prior to tMCAO. Flow cytometric analysis confirmed that the anti-CD122 mAb depleted CD8^+^CD122^+^ T cells (Figure 1D) without statistically significantly affecting the total numbers of T cells, B cells, monocytes, or dendritic cells (DCs) in the blood (Supplemental Figure 3A). The number of circulating neutrophils increased in CD8^+^ TRL–depleted mice 3 days after tMCAO (Supplemental Figure 3A). Immunostaining confirmed that the anti-CD122 mAb abolished the CD8^+^CD122^+^ T cell infiltration of the ischemic brain (Supplemental Figure 1C).

The isotype IgG– and anti-CD122 mAb–treated mice exhibited comparable regional cortical blood flow (CBF) during tMCAO and reperfusion (Figure 1E). The temporal evolution of ischemic brain injury was observed by longitudinal T2-weighted MRI scans at 1, 3, and 14 days after tMCAO in the same cohort of mice (Figure 1F). Anti-CD122 mAb–treated mice showed larger brain infarcts and more severe brain edema than IgG-treated mice from 1 to 14 days after tMCAO (Figure 1, G and H, and Supplemental Figure 2, A–D). These data indicate that endogenous CD8^+^ TRLs are critical in restricting ischemic brain injury from early stages (Supplemental Figure 2, B and C) to at least 2 weeks (Supplemental Figure 2D) after stroke. To confirm the early neuroprotective effects of CD8^+^ TRLs against stroke, FACS-isolated CD8^+^CD122^+^CD49d^lo^ TRLs were adoptively transferred to CD8^+^ TRL–depleted mice 2 hours after tMCAO. CFSE-labeled CD8^+^ TRLs were detected in the blood 24 hours after adoptive transfer (Figure 1I). Replenishment of CD8^+^ TRLs completely reversed the detrimental effects of anti-CD122 mAb on brain infarction 3 days after tMCAO (Figure 1J).

The protective role of CD4^+^ Tregs in ischemic stroke has been widely reported (2, 3, 18). Our data show that depletion of CD4^+^ Tregs with anti-CD25 mAb (100 μg) increased infarction and neuronal death at a relatively delayed stage (7 days) after tMCAO, in contrast to the early detrimental effects of anti-CD122 mAb (Figure 1K and Supplemental Figure 3B). Consistent with the increased infarct, anti-CD122 mAb–treated mice exhibited worse sensorimotor deficits 3–7 days after tMCAO, as determined by the rotarod test, adhesive removal test, and foot-fault test (Figure 1, L–N). The worsening in sensorimotor deficits was also observed in anti-CD25 mAb–treated mice but delayed until 5–7 days after tMCAO (Figure 1, L–N). Anti-CD122 mAb treatment had no effect on sensorimotor functions in sham mice (Supplemental Figure 3C). Combined injections of anti-CD122 and anti-CD25 mAbs augmented infarct 3 days after tMCAO (Supplemental Figure 3D), an effect comparable to anti-CD122 mAb injection alone (Figure 1, J and K). These results suggest that CD8^+^ TRL depletion deteriorates brain infarct regardless of the status of CD4^+^ Tregs in the early injury phase.

Consistent with previous studies (19), anti-CD122 mAb resulted in the depletion of CD8^+^CD122^+^ cells and NK cells (Supplemental Figure 3, A and E). However, depletion of NK cells alone with anti-NK1.1 mAb (100 μg) had no effect on infarct 3 or 7 days after tMCAO (Supplemental Figure 3F). Thus, the detrimental effects of anti-CD122 mAb are attributable mainly to depletion of endogenous CD8^+^CD122^+^ cells.

### CD8^+^ TRLs infiltrate the injured brain through the CXCL10/CXCR3 chemokine/receptor axis.

**Figure 2:**
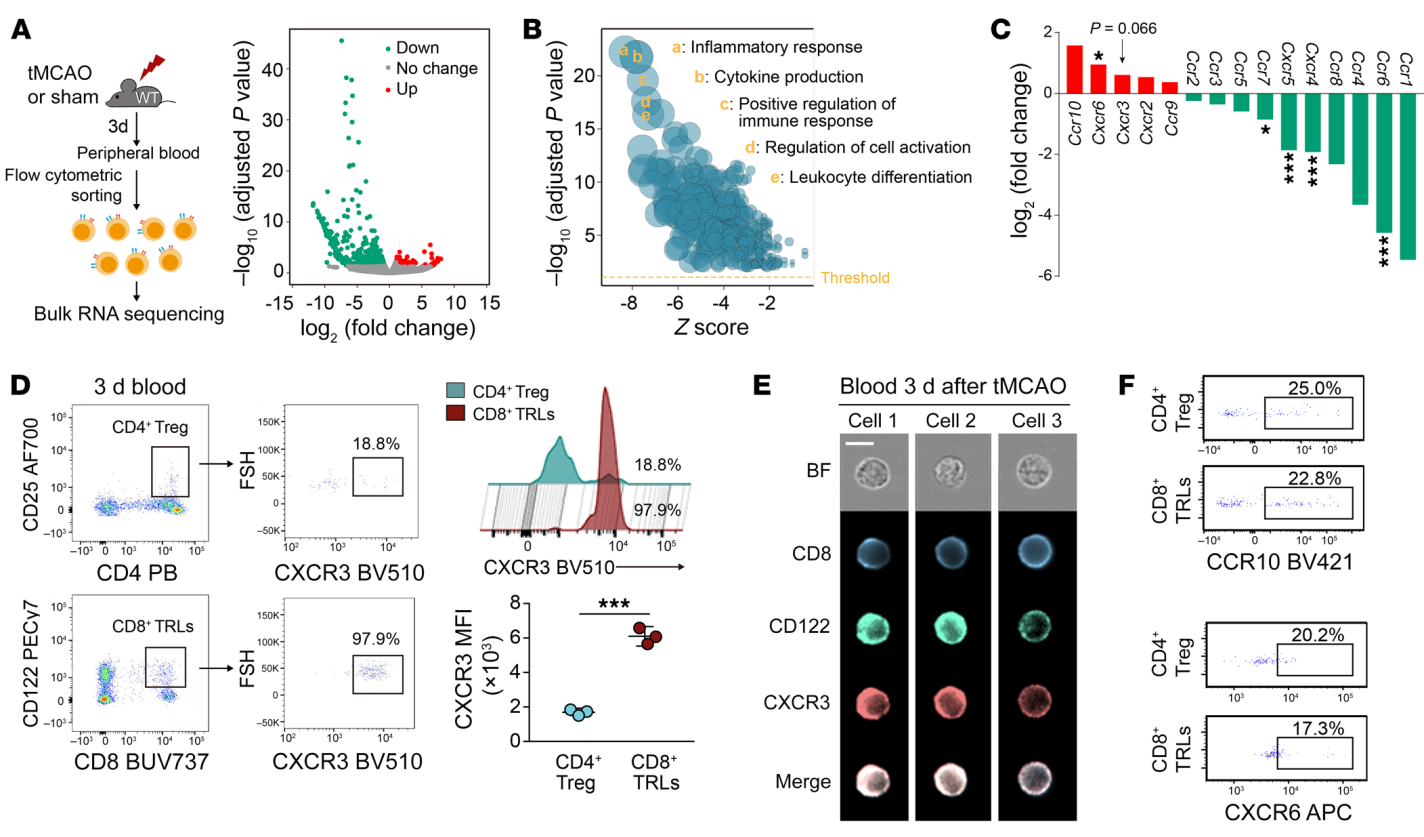
CXCR3 is upregulated in circulating CD8^+^ TRLs after stroke. (**A**) CD8^+^ TRLs were sorted from mouse blood 3 days after tMCAO or sham operation for RNA-seq analysis. Red and green dots in the volcano plot represent transcripts expressed at higher or lower levels in stroke blood versus sham blood CD8^+^ TRLs, respectively (adjusted *P <* 0.05, |fold change| > 2). (**B**) Gene Ontology analysis of the differentially expressed genes (DEGs). (**C**) The expression of several chemokine receptors on CD8^+^ TRLs from stroke blood compared to sham blood. (**D**) Flow cytometric analysis of CXCR3^+^ cells among CD8^+^ TRLs and CD4^+^ Tregs in the blood 3 days after tMCAO. The mean fluorescence intensity (MFI) of CXCR3 among CD8^+^ TRLs and CD4^+^ Tregs in the ischemic brain was quantified. *n =* 3/group. Two-tailed Student’s *t* test. (**E**) Representative ImageStream images show expression of CXCR3, CD8, and CD122 in 3 blood cells collected 3 days after tMCAO. Scale bar: 10 μm. (**F**) Flow cytometric analysis of CCR10 and CXCR6 in CD8^+^ TRLs and CD4^+^ Tregs in blood 3 days after tMCAO. The plots represent 2 independent experiments. *P < 0.05; ***P < 0.001.

The migration of circulating or other peripheral immune cells toward sites of injury relies on their expression of chemokine receptors that recognize corresponding chemokines released by injured tissue. To identify the specific receptors that mediate early intracerebral infiltration by CD8^+^ TRLs, we performed RNA sequencing (RNA-seq) analysis to compare the CD8^+^ TRLs (CD8^+^CD122^+^CD49d^lo^) isolated from blood of sham mice versus the blood of tMCAO mice, both collected 3 days after surgery (Figure 2A). Differentially expressed genes (DEGs) were identified (Figure 2A and Supplemental Table 1). Gene Ontology (GO) analysis revealed marked changes in inflammatory responses and cytokine production (Figure 2B). A panel of genes encoding chemokine receptors, including *Ccr10*, *Cxcr6*, and *Cxcr3*, was upregulated in circulating CD8^+^ TRLs after stroke (Figure 2C). Conversely, the expression of genes encoding several receptors that mediate T cell homing to lymph nodes and other peripheral organs, including *Ccr6*, *Ccr7*, and *Cxcr5*, was markedly downregulated (Figure 2C). We then compared the expression of several chemokine receptors by circulating CD8^+^ TRLs versus the CD4^+^ Tregs 3 days after stroke to identify receptors potentially important for early CD8^+^ TRL infiltration. Flow cytometric (Figure 2D) and ImageStream (Figure 2E) analyses demonstrated that expression of CXCR3 (Figure 2D) was approximately 3-fold higher on CD8^+^ TRLs compared with CD4^+^ Tregs, while expression of CXCR6, CCR10, CCR4, CCR5, CCR6, CCR7, and CXCR5 was comparable (Figure 2F and Supplemental Figure 4A).

**Figure 3:**
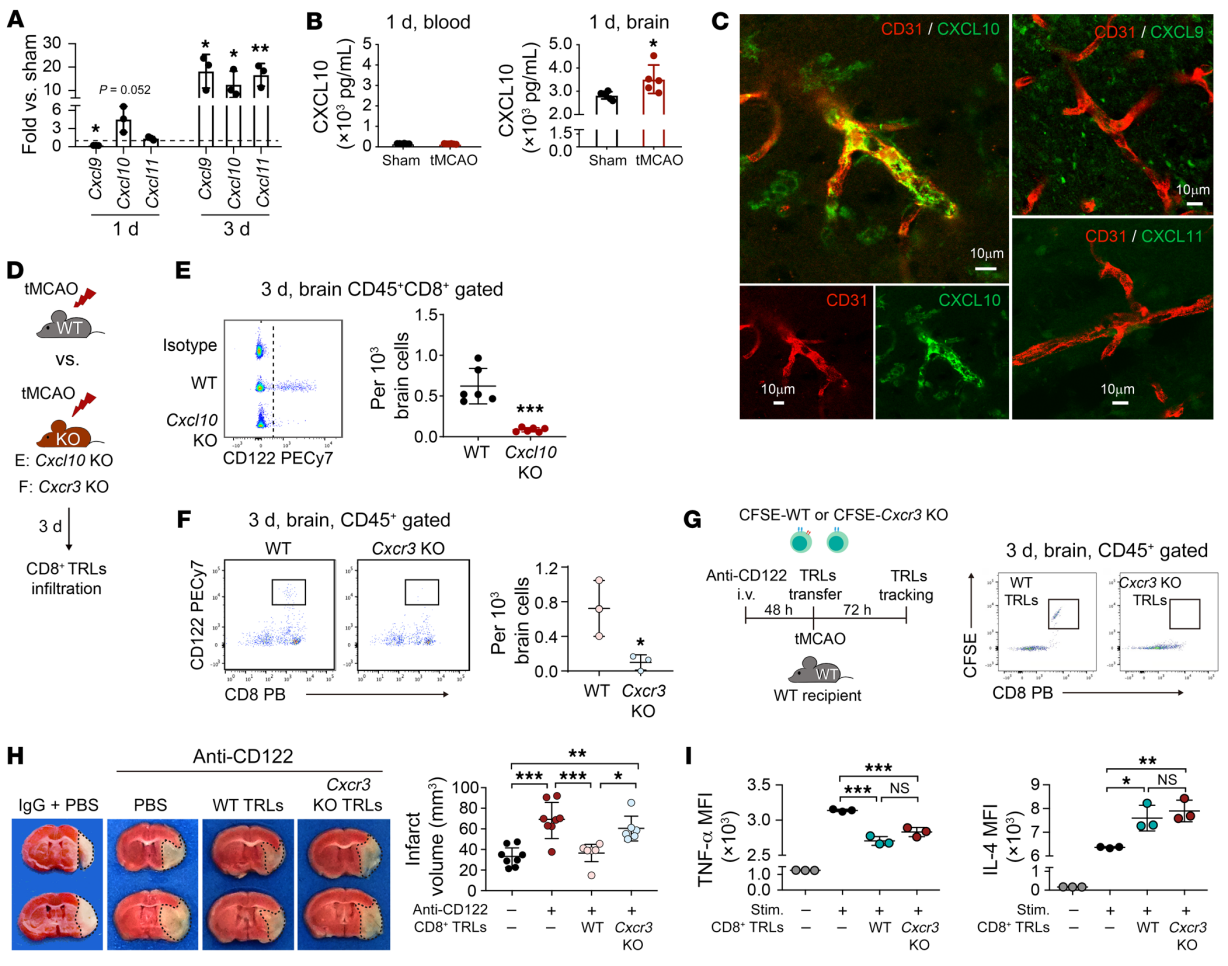
CXCR3/CXCL10-mediated brain infiltration is important for the neuroprotective effect of CD8^+^ TRLs. (**A**) RT-qPCR of CXCR3 ligand (*Cxcl9*, *Cxcl10*, and *Cxcl11*) expression in brain 1 and 3 days after tMCAO versus sham. *n =* 3/group. Two-tailed Student’s *t* test. (**B**) Expression of CXCL10 was assessed in the blood (left) and in the ischemic brain (right) by ELISA 1 day after tMCAO. *n =* 5–6/group. Two-tailed Student’s *t* test. (**C**) Expression of CXCL10, CXCL11, and CXCL9 was assessed in the CD31^+^ endothelium of the ischemic brain 3 days after stroke. Scale bars: 10 μm. (**D**–**F**) Sixty-minute tMCAO was induced in WT, *Cxcl10*-KO, or *Cxcr3*-KO mice (**D**). Brain infiltration by CD8^+^CD122^+^ TRLs was detected by flow cytometry in *Cxcl10*-KO (**E**, *n =* 6) or *Cxcr3*-KO (**F**, *n =* 3) mice 1 day after stroke. PB=pacific blue. Two-tailed Student’s *t* test. (**G** and **H**) CD8^+^ TRLs were sorted from the spleen of WT or *Cxcr3*^–/–^ mice and labeled with CFSE (1 μM). Labeled cells were injected (1 × 10^6^/mouse, i.v.) into anti-CD122 mAb–pretreated mice at 2 hours after 60-minute tMCAO. (**G**) Flow cytometry showed reduced brain infiltration by *Cxcr3*^–/–^ CFSE^+^CD8^+^ TRLs 3 days after stroke. The plots are representative of 3 animals in each group. (**H**) Infarct volumes 3 days after tMCAO. *n =* 6–8/group. One-way ANOVA and post hoc Bonferroni’s test. (**I**) The immunomodulatory effect of *Cxcr3*^–/–^ CD8^+^ TRLs was intact compared with WT CD8^+^ TRLs. CD3^+^CD25^–^CD122^–^ Teffs were sorted from the spleen of healthy donor mice and cocultured in vitro with CFSE-labeled *Cxcr3*^–/–^ or WT CD8^+^ TRLs. Cells were stimulated with PMA (80 nM) and ionomycin (1 μM) for 5 hours. The production of TNF-α and IL-4 in CFSE^–^CD3^+^ Teff cells was detected by flow cytometry. *n =* 3/group. One-way ANOVA and post hoc Bonferroni’s test. *P < 0.05; **P < 0.01; ***P < 0.001.

Furthermore, we observed marked elevations in mRNA expression of CXCR3 ligands (*Cxcl9*, *Cxcl10*, and *Cxcl11*) in brain 3 days after tMCAO (Figure 3A), whereas *Cxcl10* already displayed a statistical trend toward upregulation (*P =* 0.052) 1 day after tMCAO. ELISAs confirmed the upregulation of CXCL10 in brain lysates, but not in blood, 1 day after stroke (Figure 3B). Immunostaining demonstrated the expression of CXCL10 protein, but not CXCL9 or CXCL11, along CD31^+^ vessels in the ischemic brain 1 day after stroke (Figure 3C). CXCL10 was also expressed by astrocytes, but only weakly by microglia and not by neurons after stroke (Supplemental Figure 4B). Taken together, these data suggest that the CXCR3/CXCL10 signaling axis may promote early CD8^+^ TRL infiltration of the ischemic brain.

Supporting the brain-homing properties of CXCR3/CXCL10 for CD8^+^ TRLs after stroke, CD8^+^ TRLs failed to infiltrate ischemic brain in *Cxcl10*-KO (Figure 3, D and E) and *Cxcr3*-KO (Figure 3, D and F) mice. CD8^+^ TRLs were then prepared from WT and *Cxcr3*-KO mice, labeled with CFSE, and transferred to CD8^+^ TRL–depleted mice 2 hours after tMCAO (Figure 3G). Flow cytometric analysis showed that the WT, but not *Cxcr3*-KO CD8^+^CFSE^+^ TRLs, entered the ischemic brain 3 days after stroke (Figure 3G). The absence of *Cxcr3*-KO CD8^+^CFSE^+^ TRL infiltration led to reduced neuroprotection compared with WT CD8^+^ TRL treatment (Figure 3H). Interestingly, the loss of CXCR3 expression on CD8^+^ TRLs did not impair their immunosuppressive effects on proinflammatory cytokine TNF-α production or their ability to boost antiinflammatory cytokine IL-4 production by Teff cells in vitro (Figure 3I). These results suggest that the neuroprotective effect of CD8^+^ TRLs relies, at least partly, on their brain infiltration via the CXCR3/CXCL10 axis.

### CD8^+^ TRLs confer neuroprotection against stroke through both antiinflammatory-dependent and -independent mechanisms.

**Figure 4:**
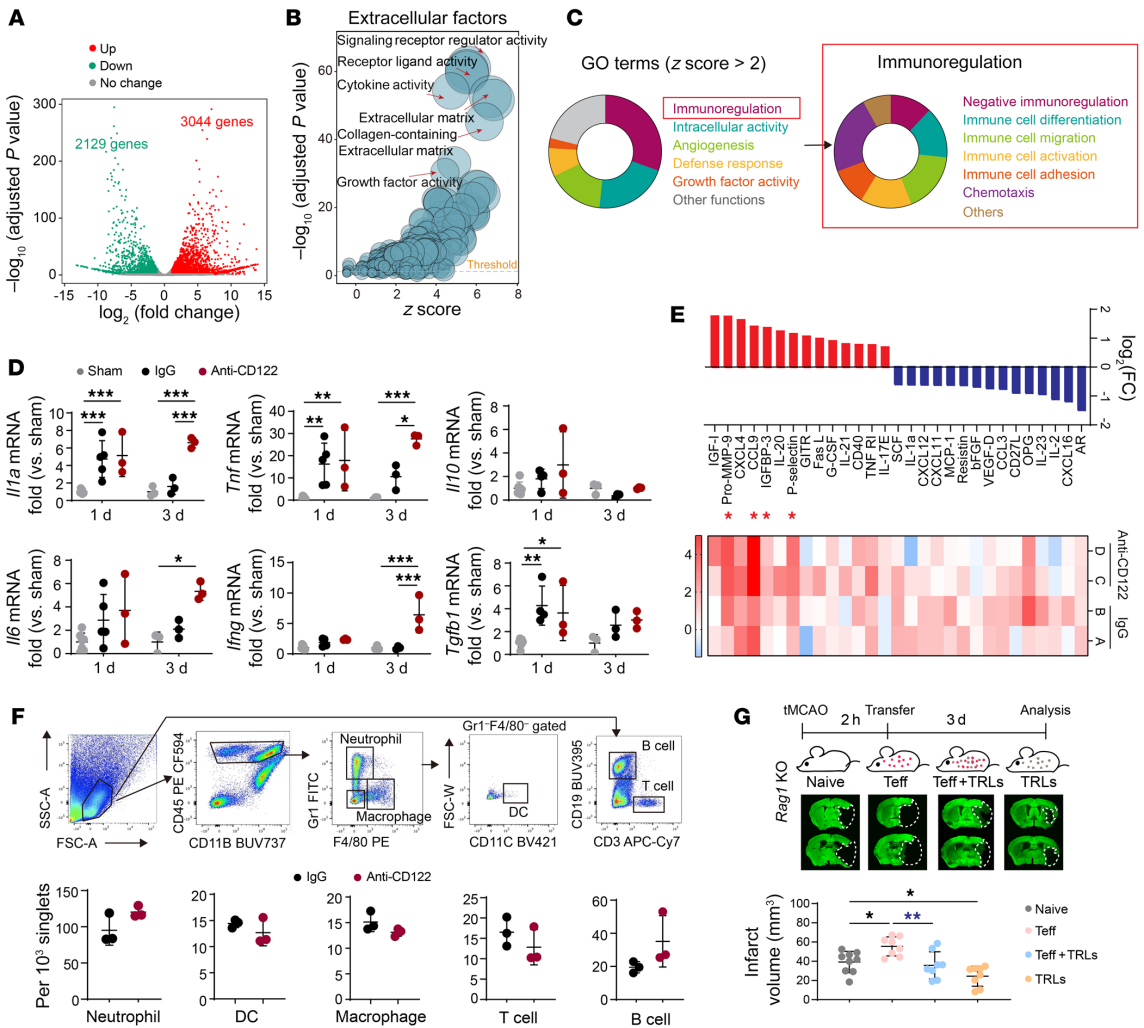
CD8^+^ TRLs confer neuroprotection after tMCAO through a combination of antiinflammatory and inflammation-independent mechanisms. (**A**–**C**) Single-cell suspensions were prepared from mouse blood and brain 3 days after sham or tMCAO. Sorted CD8^+^ TRLs were analyzed by RNA-seq. *n =* 2 in each group. (**A**) Volcano plot showing differentially expressed genes (DEGs) between brain-infiltrating TRLs and blood TRLs (adjusted *P <* 0.05, |fold change| > 2). (**B**) Gene Ontology (GO) analyses of DEGs encoding extracellular factors. (**C**) GO analyses showing the immunoregulatory function of brain-infiltrating CD8^+^ TRLs. (**D**–**F**) Mice were treated with isotype IgG (100 μg) or anti-CD122 mAb (100 μg) 2 days prior to 60-minute tMCAO. (**D**) Quantitative RT-PCR analysis for *Il1a*, *Tnf*, *Ifng*, *Il6*, *Il10*, and *Tgfb1* mRNA expression at 1 or 3 days after tMCAO. *n =* 3–7/group. Two-way ANOVA and post hoc Bonferroni’s test. (**E**) Protein array analysis 3 days after tMCAO. Heatmap and bar graphs demonstrating proteins with greater than 2-fold changes (red, upregulated; blue, downregulated) in anti-CD122–treated mice versus IgG-treated mice after stroke. *n =* 3–5/group. Red asterisks indicate proteins that were significantly upregulated with a false discovery rate (FDR) < 0.2. (**F**) Infiltration by Gr1^+^ neutrophils, CD11c^+^ DCs, F4/80^+^ macrophages, CD3^+^ T lymphocytes, and CD19^+^ B lymphocytes into the ischemic brain was quantified by flow cytometry 3 days after tMCAO. *n =* 3/group. Two-tailed Student’s *t* test. (**G**) CD3^+^CD122^–^CD25^–^ Teff cells (1 million) or PBS were transferred into *Rag1^–/–^* mice 2 hours after tMCAO, which was followed by i.v. infusion of PBS or CD8^+^ TRLs (0.5 million). Brain infarcts were quantified on MAP2-stained brain sections collected 3 days after tMCAO. *n =* 8–9/group. One-way ANOVA and post hoc Bonferroni’s test. *P < 0.05; **P < 0.01; ***P < 0.001.

We then performed RNA-seq analysis to compare the gene expression profiles of FACS-purified brain infiltrating CD8^+^ TRLs (CD8^+^CD122^+^CD49d^lo^) 3 days after tMCAO and circulating CD8^+^ TRLs 3 days after sham operation. Compared with their stroke-naive counterparts, CD8^+^ TRLs underwent genomic reprogramming after infiltration of the poststroke brain, resulting in global gene expression alterations (Figure 4A and Supplemental Table 2). To examine the regulatory impact of CD8^+^ TRLs on other cells, we performed further analyses of DEGs that encode the expression of extracellular factors (Supplemental Table 3), including cell membrane–bound and secretory factors. GO analyses of these extracellular factors revealed that brain-infiltrating CD8^+^ TRLs were characterized by enhanced expression of genes involved in cytokine activity, extracellular matrix interactions, cytokine receptor binding, and growth factor activity (Figure 4B). Large portions of the GO terms belonging to the immunoregulation category were further itemized toward negative immunoregulation and immune cell differentiation/migration/activation (Figure 4C).

The main function of CD8^+^ TRLs is immunoregulation. We therefore evaluated their influence on poststroke immune mobilization. Cerebral mRNA expression of cytokines such as *Il1a*, *Tnfa*, and *Tgfb1* increased 1 day and/or 3 days after tMCAO. CD8^+^ TRL depletion did not change the cytokine profile in brain 1 day after tMCAO, but enhanced mRNA expression of some proinflammatory genes, such as *Il1a*, *Tnf*, *Il6*, and *Ifng* 3 days after tMCAO (Figure 4D). A protein array was also performed on brain extracts 3 days after tMCAO. CD8^+^ TRL–depleted mice displayed increased expression of some proinflammatory cytokines, such as pro-MMP-9, CCL9, and P-selectin (Figure 4E). As determined by FACS, the infiltration by immune cells, such as Gr1^+^ neutrophils, CD11c^+^ DCs, F4/80^+^ macrophages, CD3^+^ T lymphocytes, and CD19^+^ B lymphocytes was not affected 3 days after stroke by CD8^+^ TRL depletion (Figure 4F).

Lymphocyte-deficient *Rag1*-KO mice were then used to confirm whether CD8^+^ TRL–mediated inhibition of Teff cells contributed to neuroprotection. CD3^+^CD122^–^CD25^–^ Teff cells (1 million) were i.v. transferred into *Rag1*-KO mice 2 hours before tMCAO, which was followed by i.v. infusion of PBS or CD8^+^ TRLs (0.5 million) (Figure 4G). The transfer of Teff cells enlarged brain infarct, whereas cotransfer of CD8^+^ TRLs significantly reduced brain infarct 3 days after tMCAO (Figure 4G). These results suggest that CD8^+^ TRLs ameliorate Teff-exacerbated brain injury after stroke. We also found that adoptive transfer of CD8^+^ TRLs reduced infarct volumes in *Rag1*-KO stroke mice without Teff transfer (Figure 4G), indicating additional Teff-independent mechanisms of neuroprotection.

### The LIFR mediates the beneficial effects of CD8^+^ TRLs after stroke.

**Figure 5:**
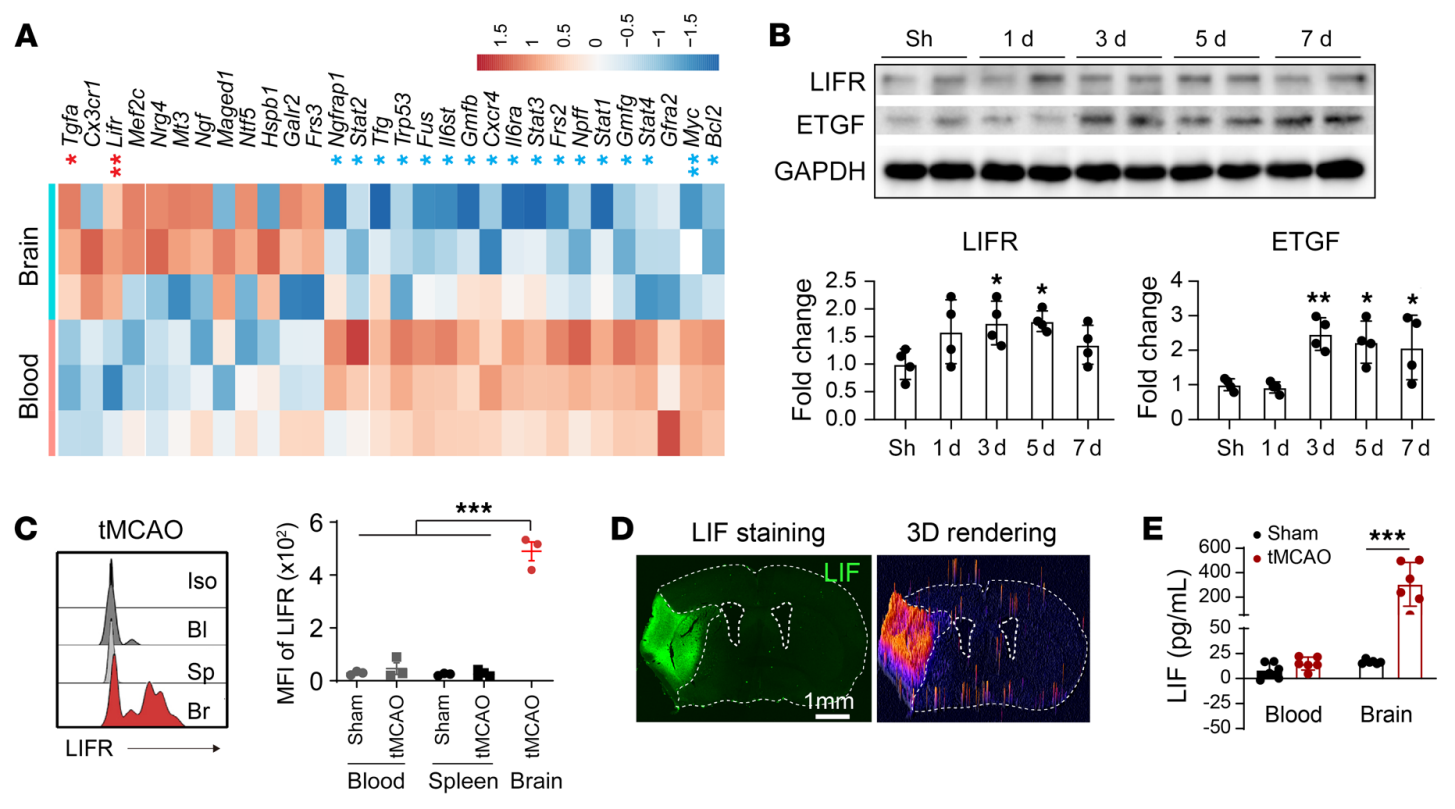
LIFR and ETGF are upregulated in CD8^+^ TRLs after stroke. (**A**) CD8^+^ TRLs from ischemic brain 3 days after tMCAO and from blood after sham operation were subjected to quantitative PCR array. The gene expression was normalized to the corresponding blood level. Heatmap showing the log_2_(fold change) for genes with >2-fold changes. *n =* 3/group. *FDR < 0.2, **FDR < 0.1 for genes upregulated (red) or downregulated (blue) in brain-infiltrating CD8^+^ TRLs. (**B**) Western blot analysis of LIFR and ETGF expression in the brain lysates collected from sham mice and 1, 3, 5, and 7 days after stroke. *n =* 4/group. One-way ANOVA and post hoc Dunnett’s test. (**C**) MFI of LIFR in CD8^+^CD122^+^ TRLs in the blood (Bl), spleen (Sp), and ipsilateral brains (Br) 3 days after tMCAO or sham operation. One-way ANOVA and post hoc Dunnett’s test. Iso, isotype control. (**D**) Immunostaining of LIF 3 days after stroke. Images are representative of 4 animals in each group. (**E**) Expression of LIF was assessed by ELISA 3 days after tMCAO. *n =* 6/group. One-way ANOVA and post hoc Bonferroni’s test. *P < 0.05; **P < 0.01; ***P < 0.001.

As CD8^+^ TRLs show immunomodulatory and, potentially, trophic effects, we used quantitative PCR arrays to compare the expression of immune modulators and neurotrophic factors in peripheral blood and brain-infiltrating CD8^+^ TRLs 3 days after tMCAO. We found that *Lifr* and *Tgfa* were significantly increased (FDR < 0.2) in brain-infiltrating CD8^+^ TRLs 3 days after tMCAO compared with peripheral CD8^+^ TRLs in sham mice, raising the possibility that CD8^+^ TRLs exert protection through these factors (Figure 5A). Western blots of ischemic tissue lysates showed increased expression of LIFR 3 and 5 days after tMCAO and increased ETGF, the protein product of *Tgfa*, 3–7 days after tMCAO (Figure 5B and Supplemental Figure 8A). Flow cytometric analysis confirmed that CD8^+^ TRLs exhibited increased expression of LIFR protein after entering the stroke brain (Figure 5C). A dramatic increase in LIF expression was found in the ischemic areas 3 days after tMCAO by immunostaining (Figure 5D) and ELISA (Figure 5E).

**Figure 6:**
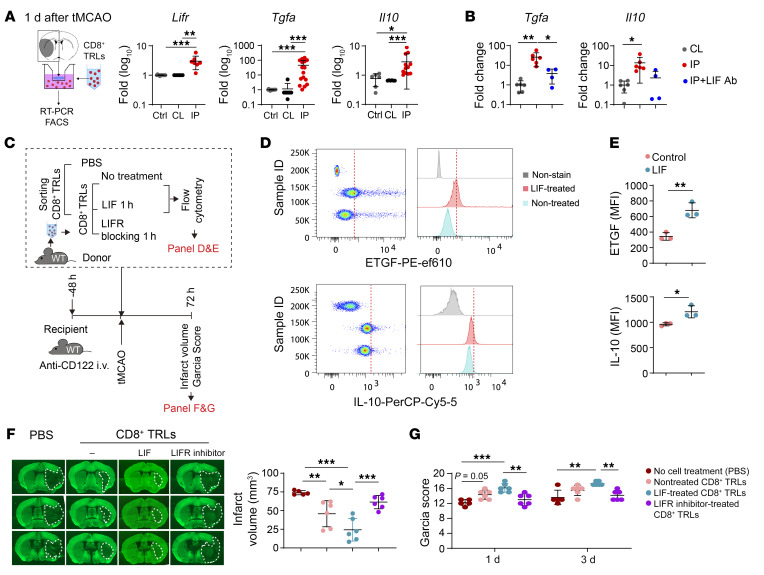
LIFR mediates the beneficial effects of CD8^+^ TRLs after stroke. (**A**) Transwell coculture system of brain slices collected 1 day after stroke and CD8^+^ TRLs from healthy spleens. RT-PCR analysis of *Lifr*, *Tgfa*, and *Il10* 24 hours after coculture. Kruskal-Wallis test and post hoc Dunn’s test. *n =* 6–16/group. (**B**) Anti-LIF antibody (60 ng/mL) was added to the CD8^+^ TRL–brain slice coculture system (as in **A**). The expression of *Tgfa* and *Il10* was measured by RT-PCR 24 hours after coculture. *n =* 4–6/group. One-way ANOVA and post hoc Bonferroni’s test. CL, contralateral brain; IP, ipsilateral brain. (**C**–**G**) Spleen-derived CD8^+^ TRLs were pretreated with LIF (100 ng/mL), LIFR inhibitor (EC359, 100 nM), or PBS for 1 hour and then injected (i.v., 1 × 10^6^ cells) into recipient mice 2 hours after tMCAO. (**D** and **E**) LIF treatment enhanced ETGF and IL-10 expression in CD8^+^ TRLs. *n =* 3/group. Two-tailed Student’s *t* test. (**F**) Quantification of MAP2 staining and (**G**) Garcia score. *n =* 5–6/group. One-way ANOVA and post hoc Bonferroni’s test. *P < 0.05; **P < 0.01; ***P < 0.001.

It is known that the LIF/LIFR signaling axis is important for the regulation of T cell fate and their response to microenvironmental cues (20). We therefore tested whether ischemia-induced activation of LIFR enhanced the expression of other protective factors by CD8^+^ TRLs. We adopted a Transwell system where FACS-purified CD8^+^ TRLs were cocultured with brain slices from the ipsilateral ischemic hemisphere and contralateral nonischemic hemisphere, respectively. Consistent with the PCR array data, coculture for 24 hours with ischemic brain slices, but not with nonischemic brain slices, significantly elevated mRNA levels of *Lifr*, *Il10*, and *Tgfa* (Figure 6A), confirming that ischemic brain tissue can induce the expression of these factors in CD8^+^ TRLs. Furthermore, blockade of LIF function by LIF-neutralizing antibody (60 ng/mL) in the coculture system abolished the elevation of *Tgfa* and *Il10* in CD8^+^ TRLs in the presence of ischemic brain slices (Figure 6B). Consistent with these observations, LIF treatment (100 ng/mL) increased IL-10 and ETGF (encoded by *Tgfa*) expression in cultured CD8^+^ TRLs (Figure 6, D and E).

To further strengthen our assessment of LIFR in CD8^+^ TRL–mediated protection, spleen-isolated CD8^+^ TRLs were pretreated with LIFR inhibitor (EC395, 100 nM), LIF (100 ng/mL), or control IgG (100 ng/mL) for 1 hour and then transferred to mice 2 hours after tMCAO (Figure 6C). Adoptive transfer of CD8^+^ TRLs reduced infarct volume (Figure 6F) and neurological deficits (Figure 6G) 3 days after tMCAO. The LIF pretreatment enhanced, while the LIFR inhibitor reduced, the protective effects of CD8^+^ TRLs.

### CD8^+^ TRLs ameliorate poststroke inflammation in an IL-10–dependent manner.

RNA-seq analyses of brain-infiltrating CD8^+^ TRLs from stroke mice compared with circulating CD8^+^ TRLs from sham mice highlighted DEGs involved in stroke-induced immunoregulation (Supplemental Figure 5A). Further pathway analyses revealed activation of the IL-10 signaling pathway in brain-infiltrating CD8^+^ TRLs (Supplemental Figure 5B). Indeed, the percentage of IL-10–producing cells among CD8^+^ TRLs was significantly increased in the ischemic brain compared with blood and spleen 3–5 days after tMCAO (Supplemental Figure 5C). The mean fluorescence intensity (MFI) of IL-10 in CD8^+^ TRLs was approximately 5-fold higher in the ischemic brain compared with CD8^+^ TRLs in the blood and spleen 5 days after tMCAO (Supplemental Figure 5D). Deficiency in IL-10 reduced the neuroprotective effects of adoptively transferred CD8^+^ TRLs in CD8^+^ TRL–depleted mice 3 days after stroke (Supplemental Figure 5, E and F). Moreover, *Il10*-KO CD8^+^ TRL–treated mice exhibited increased brain expression of IL-6, CCL1, and CCL2 after stroke compared with WT CD8^+^ TRL–treated mice (Supplemental Figure 5G). IL-6 is an important inflammation amplifier that enhances the proinflammatory effects of lymphocytes as well as myeloid cells (1). CCL1 and CCL2 serve as early chemoattractants in stroke lesions, encouraging the infiltration by immune cells (1). Upregulation of these important proinflammatory mediators in *Il10*-KO CD8^+^ TRL–treated mice suggests that IL-10 signaling mediates the antiinflammatory properties of CD8^+^ TRLs after ischemic stroke.

### CD8^+^ TRLs provide direct neuronal protection through ETGF.

**Figure 7:**
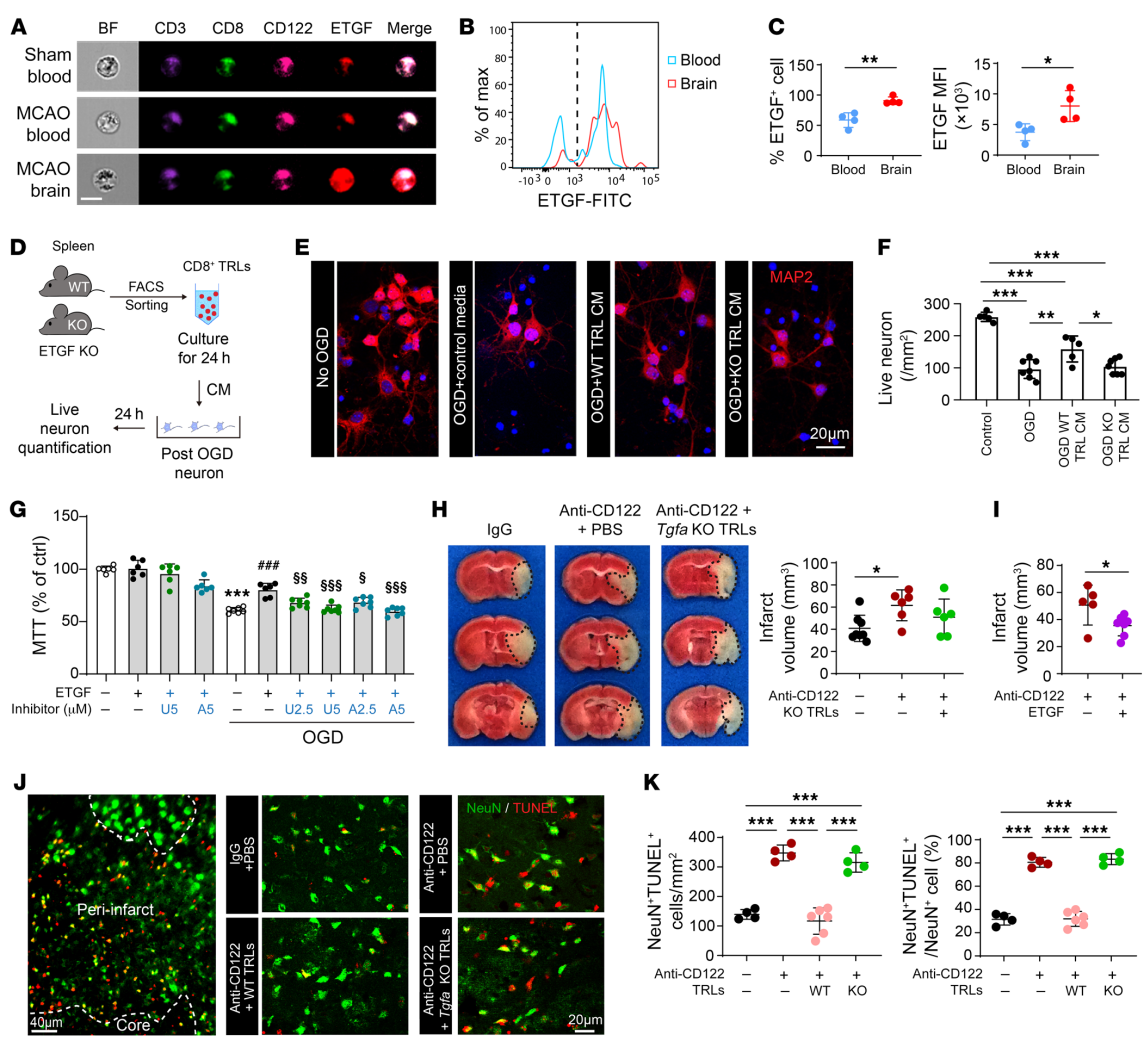
ETGF is essential for CD8^+^ TRL–afforded direct neuroprotection. (**A**) ImageStream shows expression of ETGF in CD3^+^CD8^+^CD122^+^ TRLs 3 days after stroke. Scale bar: 10 μm. (**B** and **C**) Quantification of the percentage of ETGF^+^ cells and the mean fluorescence intensity (MFI) of ETGF by flow cytometry 3 days after stroke. *n =* 4/group. Two-tailed Student’s *t* test. (**D**–**F**) Conditioned media (CM) from CD8^+^ TRLs protected neurons against 90-minute oxygen-glucose deprivation (OGD). (**D**) Experimental design. (**E**) MAP2 (red) and DAPI (blue) staining. Scale bar: 20 μm. (**F**) Numbers of MAP2^+^ live cells were quantified. *n =* 5–7/group. One-way ANOVA and post hoc Bonferroni’s test. (**G**) ETGF (40 ng/mL) protected primary neurons against 90-minute OGD in an AKT- and ERK1/2-dependent manner. AKT inhibitor VIII (A) or ERK inhibitor U0126 (U) was added at indicated concentrations together with ETGF. Cell death was quantified by MTT assay 24 hours after OGD. *n =* 6–7/group. One-way ANOVA and post hoc Bonferroni’s test. ***P < 0.001 vs. non-OGD control. ^###^*P <* 0.001 vs. OGD. ^§^*P <* 0.05, ^§§^*P <* 0.01, ^§§§^*P <* 0.001 vs. OGD + ETGF. (**H**) Mice were treated with anti-CD122 mAb (100 μg) 2 days prior to 60-minute tMCAO. CD8^+^ TRLs prepared from WT or *Tgfa*-KO mice were transferred (1 × 10^6^ cells, i.v.) into CD8^+^ TRL–depleted mice 2 hours after stroke. Infarct volumes were quantified by TTC staining 3 days after stroke. *n =* 6–8/group. One-way ANOVA and post hoc Bonferroni’s test. (**I**) ETGF (100 ng in 10 μL PBS) or an equal volume of PBS was injected intracerebroventricularly 5 minutes after reperfusion. Infarct volumes were quantified 3 days after tMCAO. *n =* 5–7/group. Two-tailed Student’s *t* test. (**J**) Representative images demonstrating TUNEL (red) colabeling with the neuronal marker NeuN (green) in peri-infarct areas. Scale bar: 20 μm. (**K**) Quantification of NeuN^+^TUNEL^+^ neurons and the percentages of NeuN^+^TUNEL^+^ neurons among all NeuN^+^ cells in the peri-infarct area. *n =* 4–6/group. One-way ANOVA and post hoc Bonferroni’s test. *P < 0.05; **P < 0.01, ***P < 0.001.

Consistent with RNA-seq results, a prominent elevation in ETGF (encoded by *Tgfa*), a member of the EGF family, was detected in brain-infiltrating CD8^+^ TRLs (Figure 7, A–C). ImageStream analyses showed higher expression of ETGF in infiltrating CD3^+^CD8^+^CD122^+^ cells 3 days after stroke (Figure 7A). FACS confirmed that the expression of ETGF was significantly higher in CD8^+^ TRLs infiltrating the ischemic brain compared with those in the blood (Figure 7, B and C). In the Transwell coculture system, the expression of ETGF was increased by 50-fold in CD8^+^ TRLs 24 hours after incubation with ischemic brain slices (Figure 6A), validating that the ischemic brain can induce intense expression of ETGF in CD8^+^ TRLs.

To confirm the direct protective effect of CD8^+^ TRLs on neurons, primary neurons were subjected to 90 minutes of oxygen-glucose deprivation (OGD) and then treated with CD8^+^ TRL–conditioned media (CM) or control media for 24 hours (Figure 7D). Administration of WT CD8^+^ TRL CM significantly preserved the number of post-OGD neurons, whereas *Tgfa*-KO CD8^+^ TRL CM failed to protect neurons against OGD (Figure 7, E and F). As proof of concept, direct ETGF treatment also protected cultured neurons against OGD through ERK1/2- and AKT-dependent signaling mechanisms (Figure 7G, Supplemental Figure 6, A–C, and Supplemental Figure 8B). In follow-up animal work, adoptive transfer of *Tgfa*-KO CD8^+^ TRLs failed to reduce infarcts in CD8^+^ TRL–depleted recipient mice 3 days after stroke (Figure 7H). Moreover, cerebroventricular injections of ETGF 2 hours after stroke significantly reduced infarct volumes in CD8^+^ TRL–depleted mice (Figure 7I). As expected, the number of dead neurons (NeuN^+^TUNEL^+^ cells) and the percentage of dead neurons (percentage of NeuN^+^TUNEL^+^ neurons among all NeuN^+^ cells) in the peri-infarct areas significantly increased in CD8^+^ TRL–depleted mice 3 days after stroke (Figure 7, J and K, and Supplemental Figure 6D). Adoptive transfer of WT CD8^+^ TRLs, but not *Tgfa*-KO CD8^+^ TRLs, reduced the number and percentage of dead neurons after stroke. Taken together, these data suggest that CD8^+^ TRLs provide direct neuronal protection through an ETGF-dependent mechanism.

### Poststroke adoptive transfer of CD8^+^ TRLs protects against acute ischemic brain injury and improves long-term recovery in young and aged mice.

**Figure 8:**
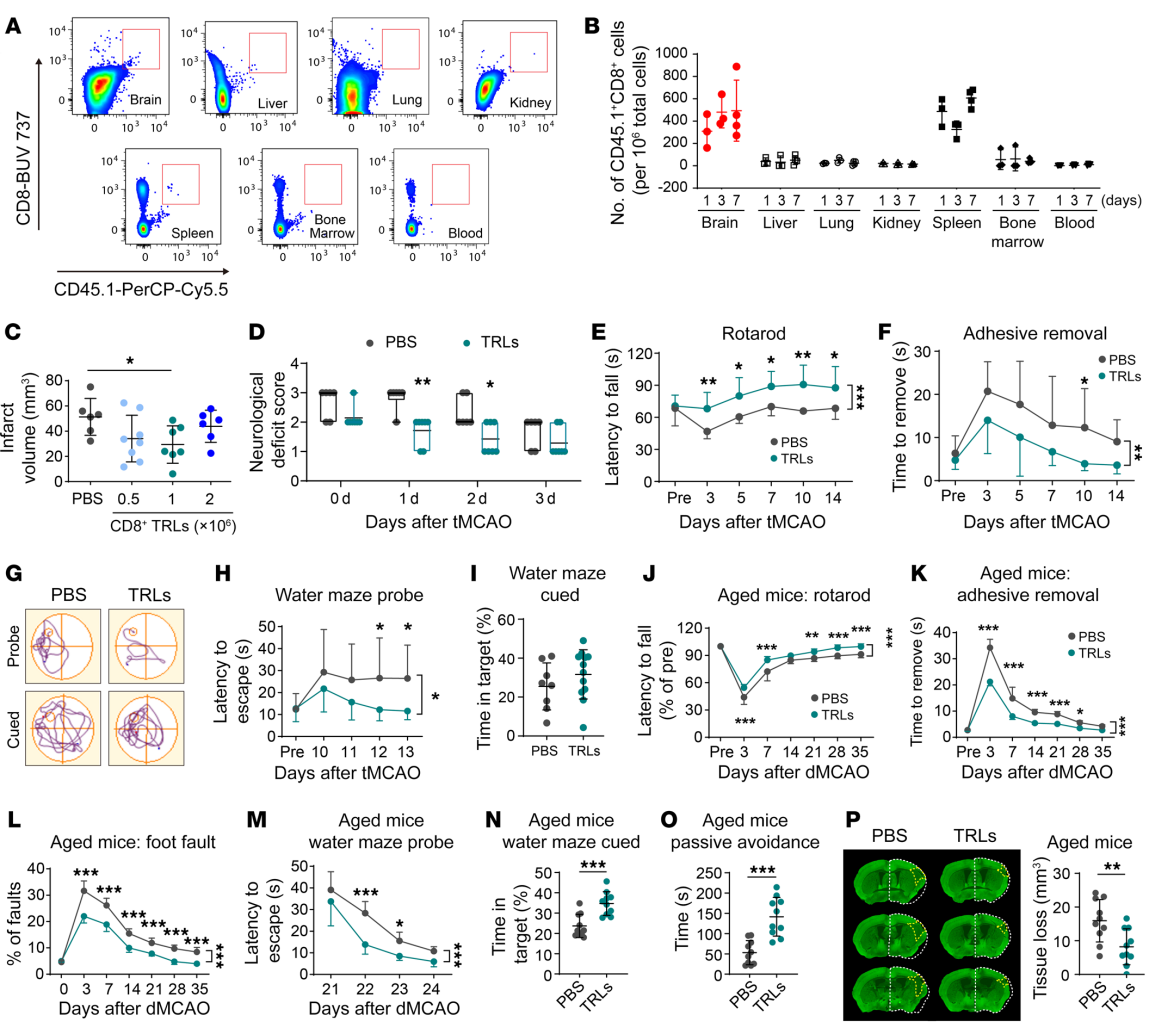
Adoptive transfer of CD8^+^ TRLs ameliorates brain infarction and improves long-term functional outcomes after stroke. CD8^+^ TRLs were isolated by FACS from the spleens of healthy young mice. (**A**–**I**) Young male stroke mice were treated intravenously with CD8^+^ TRLs or PBS 2 hours after tMCAO. (**A**) Flow cytometry to detect adoptively transferred CD45.1^+^CD8^+^ TRLs in CD45.2 congenic mice. (**B**) Quantification of CD45.1^+^CD8^+^ TRLs in various tissues 1, 3, and 7 days after tMCAO. *n =* 3–4/group. (**C**) Quantification of MAP2 staining 3 days after tMCAO in 0, 0.5 × 10^6^, 1 × 10^6^, or 2 × 10^6^ CD8^+^ TRL–treated mice. *n =* 6–8/group. (**D**) Neurological deficit score. *n =* 6–7/group. Sensorimotor dysfunction was assessed by the rotarod (**E**) and adhesive removal (**F**) tests up to 14 days after tMCAO. *n =* 8–12/group. (**G**–**I**) Morris water maze 10–14 days after tMCAO. (**G**) Representative swim paths. (**H**) Time needed to reach the hidden platform (probe phase). (**I**) Time spent in the quadrant where the platform had previously been placed was measured 14 days after tMCAO (cued phase). *n =* 8–12/group. (**J**–**P**) Aged (20-month-old) male mice were treated intravenously with 1 × 10^6^ FACS-isolated CD8^+^ TRLs or PBS 24 hours after distal MCAO (dMCAO). *n =* 10/group. Sensorimotor dysfunction was assessed by the rotarod (**J**), adhesive removal (**K**), and foot-fault tests (**L**) up to 35 days after dMCAO. (**M** and **N**) The Morris water maze test at 21–25 days after dMCAO. (**O**) Nonspatial memory was assessed 35 days after dMCAO using the passive avoidance test. Latency until entry into the dark room from the light room was recorded. (**P**) Quantification of MAP2 staining 35 days after dMCAO. The areas of contralesional hemisphere are reflected on the ipsilesional hemisphere (dashed white outlines). Yellow dashed line indicates the areas of tissue loss. *P < 0.05; **P < 0.01; ***P < 0.001. Two-tailed Student’s *t* test (**I** and **N**–**P**), Mann-Whitney test (**D**), 1-way (**C**) or 2-way ANOVA (**E**, **F**, **H**, and **J**–**M**) and post hoc Bonferroni’s test.

Given the pivotal role of CD8^+^ TRLs in determining stroke outcomes, we next assessed whether exogenous transfer of CD8^+^ TRLs could improve poststroke recovery. CD8^+^ TRLs (CD8^+^CD122^+^CD49d^lo^) were isolated from pooled spleens of healthy young donor mice (Supplemental Figure 7A). The canonical function of isolated CD8^+^ TRLs was confirmed by their suppression of the proliferation of Teff lymphocytes in cultures (Supplemental Figure 7B). tMCAO (60 minutes) was induced in young adult WT C57BL/6J males, and then mice were randomly assigned to receive adoptive transfer (i.v.) of CD8^+^ TRLs or an equal volume of PBS after 2 hours of reperfusion. Using CD45.1 congenic mice, exogenously transferred CD45.1^+^CD8^+^ TRLs (1 million cells/mouse) were detected by flow cytometry in the ipsilateral brain and spleen of stroke recipients 1, 3, and 7 days after tMCAO (Figure 8, A and B). Minimal numbers of CD45.1^+^CD8^+^ TRLs were detected in bone marrow, blood, lung, liver, and kidney (Figure 8, A and B). Adoptive transfer of 1 million CD8^+^ TRLs per mouse significantly reduced infarct volume 3 days after tMCAO (Figure 8C) and reduced the neurological deficit scores 1 and 2 days after tMCAO (Figure 8D) compared with PBS-treated mice. Transfer of CD8^+^ TRLs at 2 million per mouse or 0.5 million per mouse failed to confer statistically significant protection (Figure 8C). CD8^+^ TRL treatment improved sensorimotor functions in the rotarod and adhesive removal tests up to 14 days after tMCAO (Figure 8, E and F). In the Morris water maze, CD8^+^ TRLs improved spatial learning in stroke recipients, as indicated by reduced latency to locate the hidden platform during the probe test (Figure 8, G and H). Spatial memory, as indicated by the time in target quadrant during the cued test (Figure 8I) and swimming speed (not shown) were not affected.

To determine whether the therapeutic potential of CD8^+^ TRLs is relevant to different stroke populations in the clinic, we evaluated the therapeutic effect of CD8^+^ TRLs in aged mice using the model of distal middle cerebral artery occlusion (dMCAO) (Supplemental Figure 7E), which mimics the clinical condition of ischemic stroke patients who are unqualified for recanalization therapy. First, we evaluated the brain infiltration by endogenous CD8^+^ TRLs at 3 and 5 days after stroke in aged (20-month-old) and young adult (12-week-old) mice after dMCAO and tMCAO, respectively. There was significantly less CD8^+^ TRL cell infiltration of the ischemic brain in aged mice at 3 days compared with young mice (Supplemental Figure 7D), while CD8^+^ TRL cell infiltration was comparable at 5 days in aged versus young stroke mice. These results suggest a delayed CD8^+^ TRL infiltration in aged mice with permanent stroke, thus supporting the rationale for early CD8^+^ TRL supplementation in aged stroke victims without reperfusion. Next, we evaluated the effect of CD8^+^ TRL treatment against stroke in aged mice by assessing sensorimotor and cognitive functions up to 35 days after dMCAO. CD8^+^ TRL treatment was delayed until 24 hours after induction of dMCAO to improve the clinical translatability of the regimen. Adoptive transfer of young CD8^+^ TRLs at 1 million per mouse improved sensorimotor functions of aged recipients of both sexes, as assessed by the rotarod (Figure 8J), adhesive removal (Figure 8K and Supplemental Figure 7F), and foot-fault (Figure 8L and Supplemental Figure 7G) tests. Spatial cognitive deficits, as revealed by the Morris water maze (Figure 8, M and N, and Supplemental Figure 7, H and I) test, were also ameliorated by the CD8^+^ TRL treatment in aged male and female mice. The passive avoidance test was performed to assess fear-motivated memory 35 days after dMCAO. CD8^+^ TRL–treated mice exhibited elongated latencies to enter the shock compartment compared with PBS-treated mice (Figure 8O and Supplemental Figure 7J), indicating improved nonspatial cognitive function. CD8^+^ TRL treatment also reduced brain atrophy 35 days after dMCAO in aged mice of both sexes (Figure 8P and Supplemental Figure 7K). In summary, these data demonstrate that adoptive transfer of CD8^+^ TRLs improves long-term histological and functional outcomes after stroke.

So far, we have evaluated the therapeutic effects of CD8^+^ TRLs purified exclusively from young donors. However, T cell functionality could decline with aging (21); thus, we also tested the efficacy of CD8^+^ TRLs from healthy 20-month-old male donor mice. Adoptive transfer of 1 million aged CD8^+^ TRLs per mouse significantly reduced brain infarct volume 3 days after tMCAO in young recipient male mice (Supplemental Figure 7C).

## Discussion

Emerging evidence supports the view that immune cells, such as microglia (22) and neutrophils (23, 24), exert multifaceted effects on stroke outcomes, and that these distinct functional roles are reflected in the dynamic transcriptomic signatures of cellular subpopulations. This principle of division of labor extends across innate and adaptive immune cells and determines their spatial distributions and function across time. In this study, we found that a special CD8^+^ T cell subpopulation, CD8^+^ TRLs, rapidly invades the injured brain and is essential for neuroprotection against acute ischemic brain injury. The favorable effects of the newly described TRLs can be contrasted with the detrimental effects of general CD8^+^ T cells on ischemic brain injury (25).

CD8^+^ TRLs appeared in the brain as early as 1 day after ischemia onset and their numbers remained elevated for at least 2 weeks after stroke. The early infiltration by CD8^+^ TRLs was vital to restrict the expansion of ischemic brain damage, as depletion of CD8^+^ TRLs with anti-CD122 mAb enlarged the ischemic volume and enhanced neurological deficits. It is known that the anti-CD122 mAb depletes both CD8^+^ TRLs and NK cells (19). However, the detrimental effects of anti-CD122 mAb cannot be attributed to NK cells in this study, as depletion of NK cells with anti-NK1.1 did not enlarge the brain infarct as had been observed in anti-CD122–treated stroke mice. Indeed, previous studies using a 90-minute tMCAO model reported that NK cell depletion with anti-NK1.1 is neuroprotective against stroke (26). Moreover, replenishment of the CD8^+^ TRLs reversed the enlargement of infarct by anti-CD122 mAb 3 days after tMCAO, confirming a protective role of CD8^+^ TRLs. It is impressive that the CD8^+^ TRL–mediated neuroprotection started promptly after ischemic onset, much earlier than the protection mediated by CD4^+^ Tregs. This early action agrees with previous findings that CD8^+^ TRLs provide fast non–antigen-specific immunoregulation (13, 27). Accordingly, it has been reported that CD8^+^ TRLs are better equipped to proliferate and perform immunoregulation than their CD4^+^CD25^+^ counterparts while suppressing allograft rejection (14). The temporal distinction between two types of immune cells with similar functions reveals the complexity of poststroke immunoregulation and the necessity of precise cell targeting and proper timing of immunotherapy.

CXCR3 is a chemokine receptor highly expressed by both murine and human CD8^+^ TRLs (28). A significant increase in *Cxcr3* mRNA expression as well as in expression of its ligand CXCL10 is observed in the ischemic brain (29). However, the function of the CXCR3/CXCL10 axis in stroke remains unclear. We show here that CD8^+^ TRLs express significantly higher CXCR3 compared with CD4^+^ Tregs. *Cxcr3* KO in CD8^+^ TRLs or the absence of CXCL10 in the stroke lesions impairs brain infiltration by CD8^+^ TRLs and hinders their neuroprotective properties. These results indicate that the expression of CXCR3 on CD8^+^ TRLs and brain expression of its ligand CXCL10 are critical for quick brain infiltration by CD8^+^ TRLs after stroke.

Various surface or secretory factors expressed by CD8^+^CD122^+^ TRLs are important for their immunomodulatory effects. For example, IL-10 produced by CD8^+^ TRLs suppresses the proliferation and function of Teff cells (30). CD8^+^ TRLs kill activated T cells through Fas/FasL-mediated cytotoxicity to maintain T cell homeostasis (10) and suppress allograft rejection (31). Our transcriptome analysis revealed that brain-infiltrating CD8^+^ TRLs displayed enhanced capacities for immunoregulation and elevated expression of neuroprotective/trophic factors. In particular, we found that IL-10 and ETGF collaborated to exert neuroprotective effects after stroke. While IL-10 plays critical roles in immunoregulation and mitigation of poststroke inflammation, ETGF directly protects ischemic neurons. ETGF is a trophic factor known for promotion of cell proliferation, differentiation, and development (32). The beneficial effects of ETGF in stroke have been reported. Intraventricular injections of ETGF reduced ischemic neuronal death after tMCAO (33). In addition, intranasal delivery of ETGF promoted proliferation of neural progenitors and improved neurological functions in a chronic stroke model (34). Furthermore, we recently reported protective effects of ETGF on oligodendrocyte lineage cells and white matter integrity after ischemic stroke (35). The current study has identified the production of ETGF by infiltrating CD8^+^ TRLs, an effect that is indispensable for their neuroprotective effects against stroke lesions. It is known that IL-10 and ETGF can be released from other types of immune cells or CNS cells such as microglia (36). It is therefore possible that CD8^+^ TRLs may cooperate with other immune cells to achieve neuroprotection. The reduced neuroprotective effects of *Il10*-KO and *Tgfa*-KO CD8^+^ TRLs nonetheless support the essential role of these 2 molecules in the beneficial effects of CD8^+^ TRLs in stroke brains.

A pivotal role of LIF in T cell biology has been reported (20). Specifically, LIF regulates CD4^+^ T cell lineage development by promoting the Treg lineage–specific transcription factor, Foxp3, and simultaneously represses Th17 lineage–specific genes (37). LIF-harboring nanoparticles, which release low levels of LIF into the immediate environment of the CD4^+^ T cell in a paracrine fashion, shift T cell polarity from Th17 cells to Tregs in a nonhuman primate model of immune-mediated rejection (37). The influence of LIF on CD8^+^ T cells is unknown. Our findings suggest that LIF/LIFR interactions mediate beneficial effects of CD8^+^ TRLs early after stroke. LIFR engagement promotes production of the immunomodulatory cytokine IL-10 and trophic factor ETGF, thereby driving the reprogramming of CD8^+^ TRLs toward a protective phenotype.

We found that the number of circulating and splenic CD8^+^CD122^+^ T cells decreased sharply 1 day after stroke onset, with gradual recovery over the following days, suggesting recruitment of peripheral CD8^+^CD122^+^ T cells toward the injured brain and later replenishment of peripheral pools. Whether these CD8^+^CD122^+^ T cells arise naturally after peripheral depletion or are induced in response to certain environmental cues remains to be elucidated. The mechanisms underlying the rapid activation of CD8^+^ TRLs are also incompletely understood. In the context of infection, some memory T lymphocytes can be activated in an inflammation-dependent but antigen-independent manner (13). Such bystander-activated memory CD8^+^ T cells quickly acquire a Teff cell phenotype. Some CD8^+^CD122^+^ T cells possess properties akin to memory T cells and rapidly exert regulatory functions without antigen-specific clonal expansion (13, 27). However, other studies have documented skewed T cell receptor use by CD8^+^CD122^+^ T cells (38), suggesting their constant generation after antigen recognition in the periphery. It is therefore possible that multiple mechanisms are involved in the activation of CD8^+^ TRLs in response to different stimulators or at different stages of stroke. Indeed, there is a second drop in numbers of CD8^+^CD122^+^ T cells in the blood and spleen at approximately 1 week after stroke, along with persistent elevation of numbers of brain-infiltrating CD8^+^CD122^+^ T cells, suggesting a delayed, antigen-specific function of this population in stroke recovery, but this awaits further study.

Our study is the first to our knowledge to demonstrate the function of CD8^+^ TRLs in mouse stroke models. Human CD8^+^CXCR3(CD183)^+^ T cells are believed to exert the same functions as murine CD8^+^CD122^+^ Tregs (28). A recent clinical study in 210 stroke patients and 87 healthy controls provides evidence that circulating CD8^+^CXCR3(CD183)^+^CD62L^+^ T cells may be linked to initial stroke severity (39). The authors reported that worse stroke severity at the acute stage could result in higher CD8^+^CXCR3(CD183)^+^CD62L^+^ T cell levels. Large-scale clinical studies are warranted to further elucidate the clinical significance of this cell population in stroke.

In sum, our results suggest that the early infiltration of CD8^+^ TRLs naturally limits infarct expansion in the ischemic brain and that adoptive transfer of CD8^+^ TRLs offers a potentially novel immunotherapy for stroke. This study sheds further light on mechanisms underlying the reduction of acute ischemic brain injury by CD8^+^ TRLs and the resulting functional recovery, thereby improving our mechanistic understanding of immunomodulation of stroke outcomes and accelerating breakthroughs against an intractable brain disorder that has defied effective treatment.

## Methods

### Data and code availability

Raw fastq files for each animal/sample are in the NCBI Gene Expression Omnibus database (GEO GSE201054). This study did not generate any unique code. All software and algorithms used in this study are publicly available and listed in the Methods.

### Experimental animals

C57BL/6J WT, *Rag1*-KO, *Cxcr3*-KO, *Cxcl10*-KO, *Tgfa*-KO, and *Il10*-KO mice were purchased form The Jackson Laboratory. Mice were housed in a temperature- and humidity-controlled and specific pathogen–free animal facility with a 12-hour light-dark cycle at the University of Pittsburgh. Food and water were available ad libitum. As detailed below, we followed STAIR guidelines in the design of animal experiments, including testing aged mice of both sexes, use of 2 different stroke models, evaluation of long-term functional stroke outcomes, monitoring of regional CBF, etc. Mice were randomly assigned to experimental groups and received randomized treatments using a lottery-drawing box. All efforts were made to minimize animal suffering and the number of animals used. All surgeries, treatments, and data analyses were performed by investigators blinded to animal genotypes and experimental grouping wherever feasible.

### Murine models of transient and distal cerebral ischemia

Transient cerebral ischemia was induced in 9- to 12-week-old males by intraluminal MCAO for 60 minutes, as described previously (5). Sham-operated animals underwent the same anesthesia and exposure of arteries without MCAO. Briefly, mice were anesthetized with 1.5% to 3% isoflurane in a O_2_/N_2_O mixture. An 8-0 monofilament with a silicone-coated tip was introduced into the common carotid artery, advanced to the origin of the MCA, and left in place to limit MCA blood flow for 60 minutes. dMCAO was induced in 20-month-old male or female mice as described previously (40). As stated above, our experimental procedures were performed following criteria derived from STAIR group guidelines (41) for preclinical evaluation of stroke therapeutics. Rectal temperature was monitored at 37.0°C ± 0.5°C during surgery. Regional CBF was measured in all stroke animals using laser Doppler flowmetry or a 2-dimensional laser speckle imaging system. Animals that died or did not display at least 70% reduction in regional CBF of pre-ischemia levels using laser Doppler flowmetry were excluded a priori from further experimentation. All mortality and exclusion rates are in Supplemental Table 4. Surgeries and quantification were performed by investigators blinded to animal genotypes and experimental grouping.

### Cell depletion

Anti-CD122 (clone TM-b1, 16-1222-82, eBioscience), anti-CD25 (clone PC61.5, 16-0251-38, eBioscience), or anti-NK1.1 (clone PK136, 16-5941-82, eBioscience) was administered (100 μg per mouse, i.v.) 48 hours before tMCAO to deplete CD8^+^ TLRs, CD4^+^ Tregs, or NK cells, respectively.

### TRL isolation and adoptive transfer

Splenic, inguinal, and axillary lymph nodes were harvested from uninjured mice (8–10 weeks old) and pooled together to prepare single-cell suspensions as we described previously (3). CD3^+^CD8^+^CD122^+^CD49d^lo^ TRLs were sorted using a FACSAria sorter (BD Biosciences). For in vivo studies, 1 × 10^6^ freshly isolated CD8^+^ TRLs were transferred i.v. to recipient mice 2 hours after tMCAO or 24 hours after dMCAO through the lateral tail vein or retro-orbital venous sinus. Control mice received an equivalent volume of PBS.

### Primary cortical neuronal culture and induction of in vitro ischemia

Primary cortical neuronal cultures were prepared from C57BL/6J mouse E17 embryos as previously described (36). OGD (90 minutes) was conducted at 10 days in vitro as we described previously (36).

### In vitro TRL cultures and cocultures

Sorted TRLs were cultured in 48-well plates (0.2 million/well) coated with anti-CD3 (4 μg/mL) and anti-CD28 (5 μg/mL) in T cell culture media (RPMI1640 containing 2 mM L-glutamine, 10% FBS, 1% penicillin/streptomycin, 1 mM sodium pyruvate, and 55 μM β-mercaptoethanol) for 24 hours. CM were then collected and added to post-OGD neurons for 1 day. For brain slice–TRL cocultures, coronal brain slices (300 μm thick, ~1.10 mm anterior to bregma to ~2.06 mm posterior to bregma) were collected from ischemic mouse brains 1 day after tMCAO. Isolated TRLs in the lower chamber were incubated with brain slices in a Transwell insert in T cell culture media in the presence of soluble anti-CD3 and anti-CD28 for 1 day and then collected for further experiments. For LIFR blocking in the coculture, LIF-neutralizing antibody (AF449, R&D Systems) was added at 60 ng/mL. In some experiments, sorted CD8^+^ TRLs were treated with LIF (554004, BioLegend; 100 ng/mL, 1 hour) or LIFR inhibitor (EC359, MedChemExpress; 100 nM, 1 hour) at 37°C.

### Behavioral tests

Behavioral tests were performed by an individual blinded to experimental groups, as we described previously (5).

Neurological deficits were assessed using a 5-point-scale neurological scoring system for mice (0, no observable deficit; 1, torso flexion to right; 2, spontaneous circling to right; 3, leaning/falling to right; 4, no spontaneous movement; 5, death).

#### Rotarod and adhesive removal tests.

The latency to fall or spin around on the rungs was recorded. The adhesive removal test was performed to assess tactile responses and sensorimotor asymmetries. Tactile responses were measured by recording the time to remove the adhesive tape, with a maximum observation period of 120 seconds.

#### Foot-fault test.

Each mouse was placed on a stainless steel grid floor (20 cm × 40 cm with a mesh size of 4 cm^2^) elevated 1 m above the floor and videotaped. The number of errors (when the animals misplaced a forelimb such that it fell through the grid) was recorded for a 1-minute-long observation period.

#### Garcia score.

The modified Garcia score system was established to access sensorimotor function. An observer blinded to the experimental group evaluated the performance of each mouse after surgery. The score consists of 6 tests: spontaneous activity, body proprioception, vibrissae touch, limb symmetry, climbing, and forelimb walking with scores of 0–3 for each test (maximum score of 18).

Cognitive function was analyzed using the Morris water maze test, as described previously (42). In the learning test, 3 trials were performed on each day. The time spent to reach the platform was recorded to reflect spatial learning capability. In the memory test, the platform was removed and a single 60-second probe trial was conducted. Time spent in the goal quadrant (where the platform was previously located) was recorded to reflect spatial memory.

#### Passive avoidance test.

A chamber was divided into a light room and a dark room, with a gate between the two. Stainless steel grids were placed on the floor of the dark room to produce a mild foot shock. In the training phase, the gate was opened. The mouse was placed in the light room and allowed to enter the dark room. Once the mouse was entirely in the dark room, the gate was closed, and an electric shock (0.25 mA, 2 seconds) was delivered to the paws. The latency until the animal escaped from the dark room was recorded. One day after training, foot shock retention tests were performed to evaluate fear memory. Each mouse was placed in the light room with the gate opened, and the latency until entry into the dark room from the light room was recorded (up to 300 seconds). No electrical stimulation was applied in the test period.

### Measurement of tissue loss

Coronal brain sections (25 μm) were sliced on a freezing microtome and 6 equally spaced coronal brain sections encompassing the MCA territory (~1.10 mm anterior to bregma to ~2.06 mm posterior to bregma) were stained with an anti-MAP2 mAb (MAB3418, Millipore; 1:500). In some experiments, brain infarct was assessed immediately after sacrifice with 2% 2,3,5-triphenyltetrazolium chloride (TTC) (T8877, Sigma-Aldrich) dissolved in sterile saline. Area of tissue loss was calculated as the area of the contralateral hemisphere minus the noninfarcted area of the ipsilateral hemisphere. Brain infarct volume or volume of tissue loss was determined by multiplying the area of tissue loss by the distances between selected brain sections.

### Immunohistochemistry and image analysis

Coronal brain sections were subjected to immunofluorescence staining as we described previously (40). The following primary antibodies were used: mouse anti-NeuN (MAB377, MilliporeSigma; 1:500); goat polyclonal anti-Iba1 (Ab5076, Abcam; 1:500); rat polyclonal anti-CD31 (550274, BD Bioscience; 1:500); rabbit polyclonal anti-CXCL9 (701117, Thermo Fisher Scientific; 1:200); goat polyclonal anti-CXCL10 (Alexa Fluor 466, R&D Systems; 1:200); goat polyclonal anti-CXCL11 (Alexa Fluor 572, R&D Systems; 1:200); goat polyclonal anti-LIF (AB449-NA, R&D Systems; 1:500); and rabbit polyclonal anti-GFAP (Z0334, Dako; 1:500). For neuronal apoptosis analysis, TUNEL staining was performed after NeuN labeling according to instructions from the manufacturer (In Situ Cell Death Detection Fluorescein, 11684795910, Roche Diagnostics). All secondary antibodies (Jackson ImmunoResearch Laboratories) were diluted at 1:1000. Confocal microscopy (FluoView FV1000, Olympus) was used to capture images.

Image analysis was performed on 1 or 2 randomly selected microscopic fields in the peri-infarct areas of each section, and 2 sections covering the infarct area were assessed for each mouse brain. The images were loaded into Image J (NIH) and manually quantified by an independent observer blinded to grouping. Positively stained cells were electronically labeled with the software to avoid duplicate counting. Data are expressed as mean numbers of cells per square millimeter in the peri-infarct area. The infarct area was identified as the region in which the majority of DAPI-stained nuclei were shrunken and by the loss of NeuN or MAP2 staining. The peri-infarct area was defined as the tissue that covers a radial distance of 200 to 300 μm from the border of the infarct.

### Flow cytometry

Animals were euthanized and perfused with cold saline. Brains were dissected and the ipsilateral (left) and contralateral (right) hemispheres were collected. Brain homogenates were prepared with the Neural Tissue Dissociation Kit (T) (Miltenyi Biotec) using a gentleMACS dissociator with heaters (Miltenyi Biotec) following the manufacturer’s instructions, as we described previously (5). Spleen and blood cells were prepared as described previously (3). Fluorochrome compensation was performed with single-stained UltraComp eBeads (01-1111-41, Thermo Fisher Scientific). Flow cytometric analyses were performed on the LSRII flow cytometer (BD Biosciences). Data analyses were performed using FlowJo software. Antibodies used for flow cytometry are listed in the Supplemental Materials.

### ImageStream

Single-cell suspensions from brains or PBMCs were stained with an antibody cocktail. The following antibodies were used: anti-CD3–FITC (11-0031, Thermo Fisher Scientific; 1:400), anti-CD8–eFluor 450 (48-0081, Thermo Fisher Scientific; 1:400), anti-CD122–PECy7 (25-1222, Thermo Fisher Scientific; 1:400), anti-CXCR3–BV510 (745033, BD Biosciences; 1:200), and rabbit anti–mouse ETGF (ab9585, Abcam; 1:400) subsequently bound with anti-rabbit–Alexa Fluor 488. Fluorochrome compensation was performed with single-stained OneComp eBeads (01-1111-41, Thermo Fisher Scientific). ImageStream analysis was performed using an Amnis Imaging Flow Cytometer (MilliporeSigma). Data analysis was performed using IDEAS software (MilliporeSigma).

### MRI scanning and analyses

T2-weighted images were acquired and quantified by a blinded observer, as we described previously (5). Mice were anesthetized through a nose cone with 1%–2% isoflurane delivered in air/O_2_ (2:1). MRI was performed on a 9.4 T/30-cm AVIII HD spectrometer (Bruker Biospin) equipped with a 12 cm high-performance gradient set, using an 86 mm quadrature RF transmit volume coil, a 2-channel receive surface RF coil, and Paravision 6.0.1. A T2-weighted RARE sequence was used, with the following parameters: repetition time (TR)/echo time (TE) = 4000/40 ms, field of view (FOV) = 20 × 20 mm, acquisition matrix = 256 × 256, 21 slices with a slice thickness of 0.5 mm, 4 averages, and a RARE factor = 8. The infarct volume was determined by manual segmentation using DSI Studio software (http://dsi-studio.labsolver.org/). Brain edema and infarct were measured from MRI coronal slices. Brain infarct was identified by means of high signal on the T2 images. Percentage brain edema was calculated based on the following equation: ([ipsilateral hemisphere volume – contralateral hemisphere volume]/[contralateral hemisphere volume]) × 100.

### Real-time PCR

Total RNA was isolated from brains (ischemic hemisphere or sham brain) with the RNeasy Lipid Tissue Mini kit (74804, QIAGEN). Total RNA of sorted CD8^+^ TRLs was isolated using the RNeasy Mini Kit (74106, QIAGEN) according to the manufacturer’s instructions. RNA (1 μg) was used to synthesize the first strand of cDNA using the Superscript First-Strand Synthesis System for RT-PCR (11752, Invitrogen) according to the manufacturer’s protocols. The program for reverse transcription was 25°C for 10 minutes, 50°C for 30 minutes, 85°C for 5 minutes, and 4°C maintain. PCR was performed on the Opticon 2 Real-Time PCR Detection System (Bio-Rad) using corresponding primers (Supplemental Table 5) and SYBR Green PCR Master Mix (330503, QIAGEN). The program for real-time PCR was 95°C for 15 minutes, (94°C for 20 seconds, 59°C for 30 seconds, 72°C for 30 seconds) for 40 cycles, melting curve from 50°C to 92°C, read every 0.2°C, hold 2 seconds, incubate at 8°C. The cycle time values of each target were normalized to that of *Gapdh* in the same sample as an internal control. The expression levels of the mRNAs are reported as fold change versus sham brain, WT CD8^+^ TRLs, or vehicle-treated CD8^+^ TRLs.

### RNA-seq

RNA extraction, library preparation, and sequencing were performed at the Health Sciences Sequencing Core at UPMC Children’s Hospital of Pittsburgh, as previously described (43). CD8^+^CD122^+^CD49d^lo^ TRLs (500–1000 cells/sample) were isolated by FACS into a plate with lysis buffer. cDNA was generated from cell lysates using the SMART-Seq Ultralow Input RNA Kit (Takara Bio), according to the manufacturer’s instructions. The cDNA product was checked by an Agilent Fragment Analyzer system for quality control. The sequencing library was constructed by following the Illumina Nextera XT Sample Preparation Guide. One nanogram of input cDNA was tagged and fragmented (“tagmented”) and amplified using the Illumina Nextrera XT kit (15031942). Sequence libraries of each sample were finally equimolarly pooled and sequenced on an Illumina Nextseq 500 system, using a paired-end 75-bp strategy. All RNA-seq data are deposited in the NCBI GEO database.

### RNA-seq data analysis

RNA-seq data were analyzed as described previously (43). Preprocessing of the RNA-seq data was completed using the Tophat-cufflinks workflow (http://cole-trapnell-lab.github.io/cufflinks/). Fastq files were quality controlled using FastQC (http://www.bioinformatics.babraham.ac.uk/projects/fastqc/). All samples passed quality control criteria. After cleaning up adapter contamination and low-quality regions, reads were mapped to the GRCm38 (mm10) mouse genome using Tophat (https://ccb.jhu.edu/software/tophat/index.shtml). We then applied Cufflinks9 (2.2.1) (http://cole-trapnell-lab.github.io/cufflinks/) to estimate gene and isoform abundance. Genes were identified by Ensembl ID (https://www.ensembl.org). Read counts and fragments per kilobase of transcript per million mapped reads (FPKM) was calculated using R/Bioconductor.

### Differential expression analysis

The R package DESeq2 (v1.30.1) (http://bioconductor.org/packages/ DESeq2) was used for normalizing the counts and performing differential expression analysis between 2 conditions. The resulting *P* values were adjusted using the Benjamini and Hochberg approach for controlling the FDR. Heatmaps were generated using the R package pheatmap (v1.0.12) (https://rdrr.io/cran/pheatmap/). Volcano plots were generated using the R package ggplot2 (v3.3.3) (https://rdrr.io/cran/cmocean/man/ggplot2.html).

### GO enrichment

Functional enrichment analysis was performed using the online tool Metascape (https://metascape.org/gp/index.html) (44). Significantly overrepresented GO terms with a minimum count of 3, and an enrichment factor (the ratio between the observed counts and the counts expected by chance) larger than 1.5 were downloaded from the Metascape. R package GOplot (https://wencke.github.io/) was used to calculate *z* score. A term was considered significantly enriched with a Benjamin-Hochberg–adjusted *P* value of less than 0.05 and |*z* score| of greater than 2. Bubble plots were generated using the R package GOplot.

### Ingenuity Pathway Analysis

DEGs identified by DEseq2 were submitted to Ingenuity Pathway Analysis for functional analysis using the Ingenuity Knowledge Base (Qiagen Bioinformatics). The fold change and adjusted *P* value for each gene were used to perform the core analysis. Pathways were considered significantly enriched with an adjusted *P* value of overlap less than 0.01 and an activation *z* score greater than 2 (predicted to be activated) or less than –2 (predicted to be inhibited).

### Statistics

Sample sizes for animal studies were determined by power analyses, allowing us to detect differences with 80% power at the expected effect sizes and with the expected variance observed in that assay. The expected effect sizes in our work range from 0.6 to 3 — depending on the dependent variable of interest. The power analyses were initially based on pilot studies and an extensive body of literature in preclinical stroke. The type I error rate was limited using an α level of 5%, with Bonferroni’s adjustment for pairwise comparisons. Results are presented as mean ± standard deviation (SD). GraphPad Prism software (v9.0.0) was used for statistical analyses. The 2-tailed Student’s *t* test (equal variances) or Welch’s *t* test (unequal variances) was used for comparison of 2 groups for continuous variables with normal distributions. The Mann-Whitney *U* rank-sum test was used for variables with non-normal distributions. The differences among multiple groups were analyzed using 1-way or 2-way analysis of variance (ANOVA) or the nonparametric Kruskal-Wallis test. Differences in means across groups with repeated measurements over time were analyzed using repeated measures ANOVA. When the ANOVA showed significant differences, pairwise comparisons between means were tested by post hoc Bonferroni (comparisons between all conditions), Dunnett (all conditions compared with a control group), or Dunn’s (following Kruskal-Wallis) tests. In all analyses, a *P* value of less than 0.05 was considered statistically significant. Details of all statistical analyses are in Supplemental Table 6.

### Study approval

All animal procedures were approved by the University of Pittsburgh Institutional Animal Care and Use Committee and performed in accordance with the NIH *Guide for the Care and Use of Laboratory Animals* (National Academies Press, 2011). All animal data are reported in accordance with the Animal Research: Reporting In Vivo Experiments (ARRIVE) 2.0 guidelines (45).

## Author contributions

WC, LS, JZ, CD, QY, JL, CJ, TY, WZ, HP, FY, SH, and ZS performed in vivo experiments and data analyses. FX, XD, and TY performed cell culture experiments and data analysis. WC, LS, and ZS performed bulk RNA-seq and data analysis. TKH performed the MRI and contributed to editing of the manuscript. LS and FX analyzed the MRI data. WC, LS, FY, and XH wrote the manuscript. YS, AWT, RKL, and JC provided scientific feedback and revised the manuscript. JC and XH conceptualized, designed, and supervised the study.

## Supplementary Material

Supplemental data

Supplemental table 1

Supplemental table 2

Supplemental table 3

## Version 1 08/01/2022

Electronic publication

## References

[1] An C (2014). Molecular dialogs between the ischemic brain and the peripheral immune system: dualistic roles in injury and repair. Prog Neurobiol.

[2] Liesz A (2009). Regulatory T cells are key cerebroprotective immunomodulators in acute experimental stroke. Nat Med.

[3] Li P (2013). Adoptive regulatory T-cell therapy protects against cerebral ischemia. Ann Neurol.

[4] Ren X (2011). Regulatory B cells limit CNS inflammation and neurologic deficits in murine experimental stroke. J Neurosci.

[5] Shi L (2021). Treg cell-derived osteopontin promotes microglia-mediated white matter repair after ischemic stroke. Immunity.

[6] Ito M (2019). Brain regulatory T cells suppress astrogliosis and potentiate neurological recovery. Nature.

[7] Endharti AT (2011). CD8^+^CD122^+^ regulatory T cells (Tregs) and CD4^+^ Tregs cooperatively prevent and cure CD4^+^ cell-induced colitis. J Immunol.

[8] Lee YH (2008). Essential role of CD8^+^CD122^+^ regulatory T cells in the recovery from experimental autoimmune encephalomyelitis. J Immunol.

[9] Liu J (2015). CD8(+)CD122(+) T-cells: a newly emerging regulator with central memory cell phenotypes. Front Immunol.

[10] Akane K (2016). CD8^+^CD122^+^CD49d^low^ regulatory T cells maintain T-cell homeostasis by killing activated T cells via Fas/FasL-mediated cytotoxicity. Proc Natl Acad Sci U S A.

[11] Li S (2014). A naturally occurring CD8(+)CD122(+) T-cell subset as a memory-like Treg family. Cell Mol Immunol.

[12] Suzuki H (2008). Are CD8^+^CD122^+^ cells regulatory T cells or memory T cells?. Hum Immunol.

[13] Wan N (2008). Bystander central memory but not effector memory CD8^+^ T cells suppress allograft rejection. J Immunol.

[14] Dai Z (2014). Natural CD8^+^CD122^+^ T cells are more potent in suppression of allograft rejection than CD4^+^CD25^+^ regulatory T cells. Am J Transplant.

[15] Bodhankar S (2015). Regulatory CD8(+)CD122 (+) T-cells predominate in CNS after treatment of experimental stroke in male mice with IL-10-secreting B-cells. Metab Brain Dis.

[16] Kim HJ (2015). Stable inhibitory activity of regulatory T cells requires the transcription factor Helios. Science.

[17] Saitoh O (2007). CD8^+^CD122^+^ T cells, a newly identified regulatory T subset, negatively regulate Graves’ hyperthyroidism in a murine model. Endocrinology.

[18] Wang J (2015). Activated regulatory T cell regulates neural stem cell proliferation in the subventricular zone of normal and ischemic mouse brain through interleukin 10. Front Cell Neurosci.

[19] Wang LX (2010). CD122^+^CD8^+^ Treg suppress vaccine-induced antitumor immune responses in lymphodepleted mice. Eur J Immunol.

[20] Metcalfe SM (2011). LIF in the regulation of T-cell fate and as a potential therapeutic. Genes Immun.

[21] Goronzy JJ, Weyand CM (2019). Mechanisms underlying T cell ageing. Nat Rev Immunol.

[22] Hu X (2020). Microglia/macrophage polarization: Fantasy or evidence of functional diversity?. J Cereb Blood Flow Metab.

[23] Cuartero MI (2013). N2 neutrophils, novel players in brain inflammation after stroke: modulation by the PPARγ agonist rosiglitazone. Stroke.

[24] Garcia-Culebras A (2018). Myeloid cells as therapeutic targets in neuroinflammation after stroke: Specific roles of neutrophils and neutrophil-platelet interactions. J Cereb Blood Flow Metab.

[25] Liesz A (2011). Inhibition of lymphocyte trafficking shields the brain against deleterious neuroinflammation after stroke. Brain.

[26] Gan Y (2014). Ischemic neurons recruit natural killer cells that accelerate brain infarction. Proc Natl Acad Sci U S A.

[27] Dai H (2010). Cutting edge: programmed death-1 defines CD8^+^CD122^+^ T cells as regulatory versus memory T cells. J Immunol.

[28] Shi Z (2009). Human CD8^+^CXCR3^+^ T cells have the same function as murine CD8^+^CD122^+^ Treg. Eur J Immunol.

[29] Wang X (2000). Identification and molecular characterization of rat CXCR3: receptor expression and interferon-inducible protein-10 binding are increased in focal stroke. Mol Pharmacol.

[30] Rifa’i M (2008). CD8^+^CD122^+^ regulatory T cells recognize activated T cells via conventional MHC class I-alphabetaTCR interaction and become IL-10-producing active regulatory cells. Int Immunol.

[31] Liu H (2017). Suppression of allograft rejection by CD8^+^CD122^+^PD-1^+^ Tregs is dictated by their Fas ligand-initiated killing of effector T cells versus Fas-mediated own apoptosis. Oncotarget.

[32] Fallon J (2000). In vivo induction of massive proliferation, directed migration, and differentiation of neural cells in the adult mammalian brain. Proc Natl Acad Sci U S A.

[33] Justicia C (2001). Administration of transforming growth factor-alpha reduces infarct volume after transient focal cerebral ischemia in the rat. J Cereb Blood Flow Metab.

[34] Guerra-Crespo M (2010). Intranasal administration of PEGylated transforming growth factor-alpha improves behavioral deficits in a chronic stroke model. J Stroke Cerebrovasc Dis.

[35] Dai X (2020). TGFα preserves oligodendrocyte lineage cells and improves white matter integrity after cerebral ischemia. J Cereb Blood Flow Metab.

[36] Yang Y (2017). ST2/IL-33-dependent microglial response limits acute ischemic brain injury. J Neurosci.

[37] Park J (2011). Modulation of CD4^+^ T lymphocyte lineage outcomes with targeted, nanoparticle-mediated cytokine delivery. Mol Pharm.

[38] Baek H (2016). Neuroprotective effects of CD4^+^CD25^+^Foxp3^+^ regulatory T cells in a 3xTg-AD Alzheimer’s disease model. Oncotarget.

[39] Li S (2021). Change and predictive ability of circulating immunoregulatory lymphocytes in long-term outcomes of acute ischemic stroke. J Cereb Blood Flow Metab.

[40] Liu X (2016). Interleukin-4 is essential for microglia/macrophage M2 polarization and long-term recovery after cerebral ischemia. Stroke.

[41] Saver JL, Albers GW, Dunn B, Johnston KC, Fisher M, STAIR VI Consortium (2009). Stroke Therapy Academic Industry Roundtable (STAIR) recommendations for extended window acute stroke therapy trials. Stroke.

[42] Pu H (2021). Intranasal delivery of interleukin-4 attenuates chronic cognitive deficits via beneficial microglial responses in experimental traumatic brain injury. J Cereb Blood Flow Metab.

[43] Jiang L (2020). Transcriptomic and functional studies reveal undermined chemotactic and angiostimulatory properties of aged microglia during stroke recovery. J Cereb Blood Flow Metab.

[44] Zhou Y (2019). Metascape provides a biologist-oriented resource for the analysis of systems-level datasets. Nat Commun.

[45] Percie du Sert N (2020). The ARRIVE guidelines 2.0: Updated guidelines for reporting animal research. J Cereb Blood Flow Metab.

