# Supplementary material for: Neuroprotection against ischemic stroke requires a specific class of early responder T cells in mice
Source: J Clin Invest. 2022 Aug 1;132(15):e157678. doi: 10.1172/JCI157678 (PMC9337834; doi:10.1172/JCI157678)
Supplement: Supplemental data [file jci-132-157678-s051.pdf]

Neuroprotection against ischemic stroke requires a specific class of early responder T cells in mice

Wei Cai,[1,#] Ligen Shi,[1,#] Jingyan Zhao,[1] Fei Xu,[1,2] Connor Dufort,[1] Qing Ye,[1,2] Tuo Yang,[1,2] Xuejiao Dai,[1] Junxuan Lyu,[1] Chenghao Jin,[1] Hongjian Pu,[1,2] Fang Yu,[1] Sulaiman Hassan,[1,2] Zeyu Sun, [1] Wenting Zhang,[1,2] T. Kevin Hitchens,[3] Yejie Shi,[1,2] Angus W. Thomson,[4] Rehana K. Leak,[5] Xiaoming Hu,[1,2,*] and Jun Chen[1,2,*]

[1]Pittsburgh Institute of Brain Disorders and Recovery, and Department of Neurology, School of Medicine, University of Pittsburgh, Pittsburgh, PA, USA.

[2]Geriatric Research, Education and Clinical Center, Veterans Affairs Pittsburgh Health Care System, Pittsburgh, PA, USA.

[3]Animal Imaging Center and Department of Neurobiology, School of Medicine, University of Pittsburgh, Pittsburgh, PA, USA.

[4]Starzl Transplantation Institute, Department of Surgery, and Department of Immunology, University of Pittsburgh School of Medicine, Pittsburgh, Pennsylvania, USA

[5]Graduate School of Pharmaceutical Sciences, Duquesne University, Pittsburgh, PA, USA.

# These authors contributed equally.

* Correspondence: chenj2@upmc.edu (J. C) or hux2@upmc.edu (X.H.)

Supplemental method:

Antibodies used for flow cytometry: The following antibodies were used: anti–CD45 PerCP-Cy5.5 (45-0451, Thermo Fisher Scientific, 1:400); anti–CD3 APC (17-0032, Thermo Fisher Scientific, 1:400); anti–CD3 eFluor450 (48-0031, Thermo Fisher Scientific, 1:400); anti–CD3 FITC (11-0031, Thermo Fisher Scientific, 1:400); anti-CD3 PerCP (100325, BioLegend, 1:400); anti–CD3 APC eFluor 780 (47-0032, Thermo Fisher Scientific, 1:400); anti–CD19 FITC (11-0193, Thermo Fisher Scientific, 1:400); anti-CD8 FITC (11-0081, Thermo Fisher Scientific, 1:400); anti-CD8 eFluor450 (48-0081, Thermo Fisher Scientific, 1:400); anti-CD8 BUV737 (612759, BD Biosciences, 1:400); anti-CD8 BV510 (563068, BD Biosciences, 1:400); anti-CD8 BV421 (100737, BioLegend, 1:400); anti-CD8 Pacific blue (MCD0828, Thermo Fisher Scientific, 1:400); anti-NK1.1 PE (12-5941, Thermo Fisher Scientific, 1:400); anti-NK1.1 APC (17-5941, Thermo Fisher Scientific, 1:400); anti-CD45.1 PerCP-Cy5.5 (110728, BioLegend, 1:400); anti-CD4 FITC (11-0041, Thermo Fisher Scientific, 1:400); anti-CD4 APC (17-0042, Thermo Fisher Scientific, 1:400); anti-CD4 Pacific blue (100428, BioLegend, 1:400); anti-CD25 Alexa Fluor700 (102024, BioLegend, 1:400); anti-CD122 PE-Cy7 (25-1222, Thermo Fisher Scientific, 1:400); anti-CD11b BUV737 (612801, BD Biosciences, 1:400); anti-CD11c BV421 (117329, BioLegend, 1:400); anti-Ly6G BUV395 (563978, BD Biosciences, 1:400); anti-F4/80 PE (157303, BioLegend, 1:400); anti-Foxp3 APC (17-5773, Thermo Fisher Scientific, 1:200); anti-IL-10 APC (17-7101, Thermo Fisher Scientific, 1:200); anti-IL-10 PE (12-7101, Thermo Fisher Scientific, 1:200); anti-HELIOS FITC (137214, BioLegend, 1:200); anti-CD49d BV510 (745007, BD Biosciences, 1:400); anti-CD103 APC (121413, BioLegend, 1:400); anti-CD45.1 PerCP-Cy5.5 (110728, BioLegend, 1:400); anti-LIFR PE (FAB5990P, R&D system, 1:100); anti-CXCR3 BV510 (745033, BD Biosciences, 1:200); anti-CCR6 BV421 (129817, BioLegend, 1:200); anti-CCR6 APC (129813, BioLegend, 1:200); anti-CXCR6 APC (151105, BioLegend, 1:200); anti-CCR5 PE Dazzle594 (359126, BioLegend, 1:200); anti-CCR7 BV605 (120125, BioLegend, 1:200); anti-CXCR5 PE Dazzle594 (145521, BioLegend, 1:200); anti-CCR4 PE (131203, BioLegend, 1:200); anti-CX3CR1 FITC (149019, BioLegend, 1:200). For flow cytometric staining of CCR10, anti-rabbit-BV421 (406410, BioLegend, 1:1000) was used as a secondary antibody after incubation with rabbit anti mouse CCR10 (ab1656, abcam, 1:400).

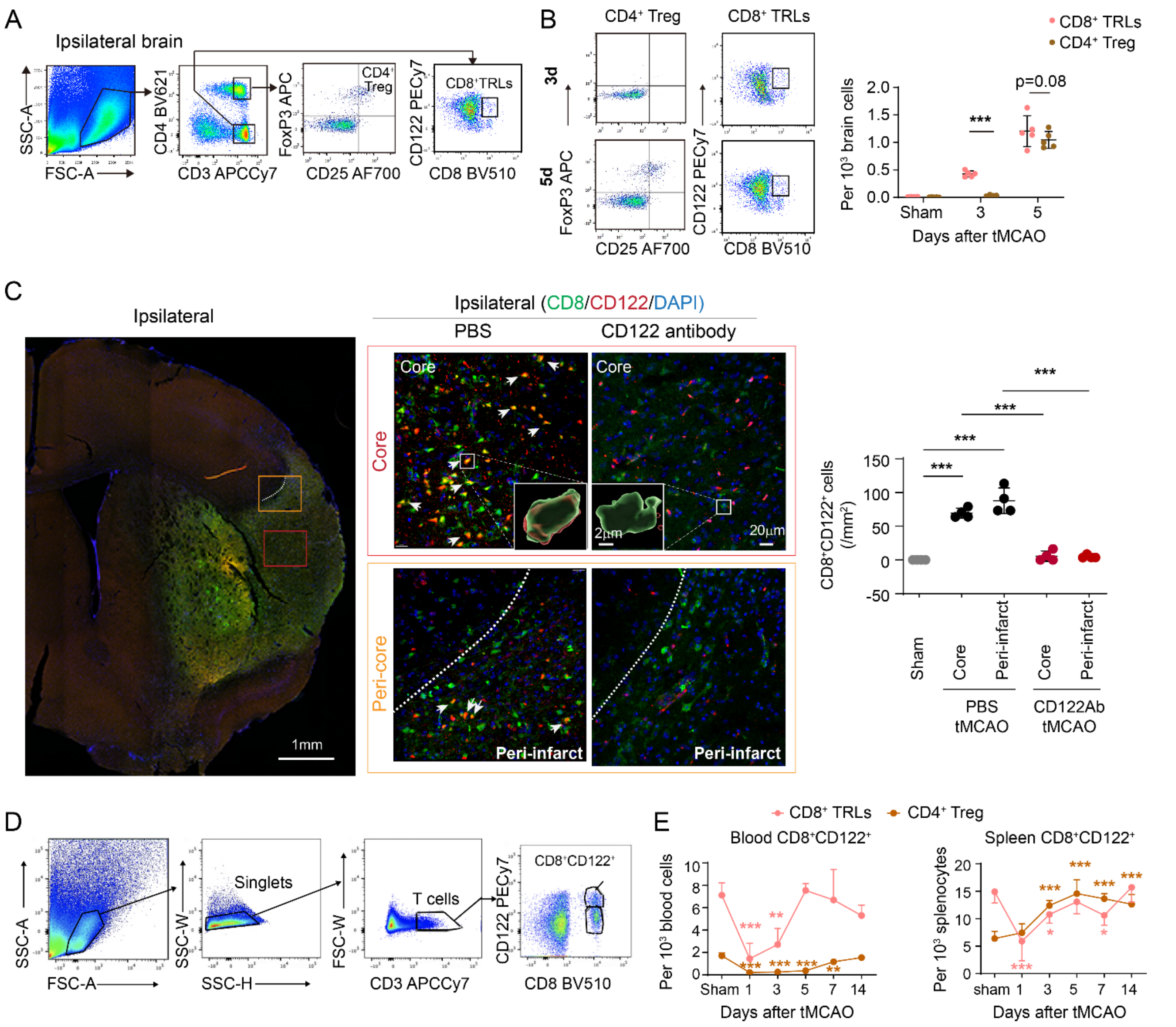

Supplemental Fig. 1. Changes in the numbers of CD8^{+}CD122^{+} TRLs after tMCAO. (A) Gating strategy for CD8^{+}CD122^{+} TRLs and CD4^{+} Tregs in the ischemic brain. **(B)** Brain infiltration of CD4^{+}CD25^{+}Foxp3^{+} Tregs and CD8^{+}CD122^{+} TRLs in sham and at 3d and 5d after tMCAO. n=4-5/group. Two-way ANOVA and *post hoc* Fisher's LSD **(C)** Triple-label immunofluorescence (CD8/CD122/DAPI) detects CD8^{+}CD122^{+} TRLs in the infarct core and peri-infarct areas (inner boarder) 5d after tMCAO. Anti-CD122 mAb treatment (100 µg, 2d prior to 60 min tMCAO) diminished the infiltration of CD8^{+}CD122^{+} TRLs into the ischemic brain. The inserts are 3D rendered of the selected cells using Imaris. The number of CD122^{+}CD8^{+} TRLs were quantified. n=4/group. One-way ANOVA and *post hoc* Bonferroni. **(D)** Gating strategy for CD8^{+}CD122^{+} TRLs in the blood and spleen. **(E)** Quantification of blood and spleen

$CD8^+CD122^+$ TRLs and $CD4^+$ Tregs at indicated time points after tMCAO. n=3-8/group. * $p<0.05$, ** $p<0.01$, *** $p<0.001$ vs. sham controls. One way ANOVA and Dunnett.

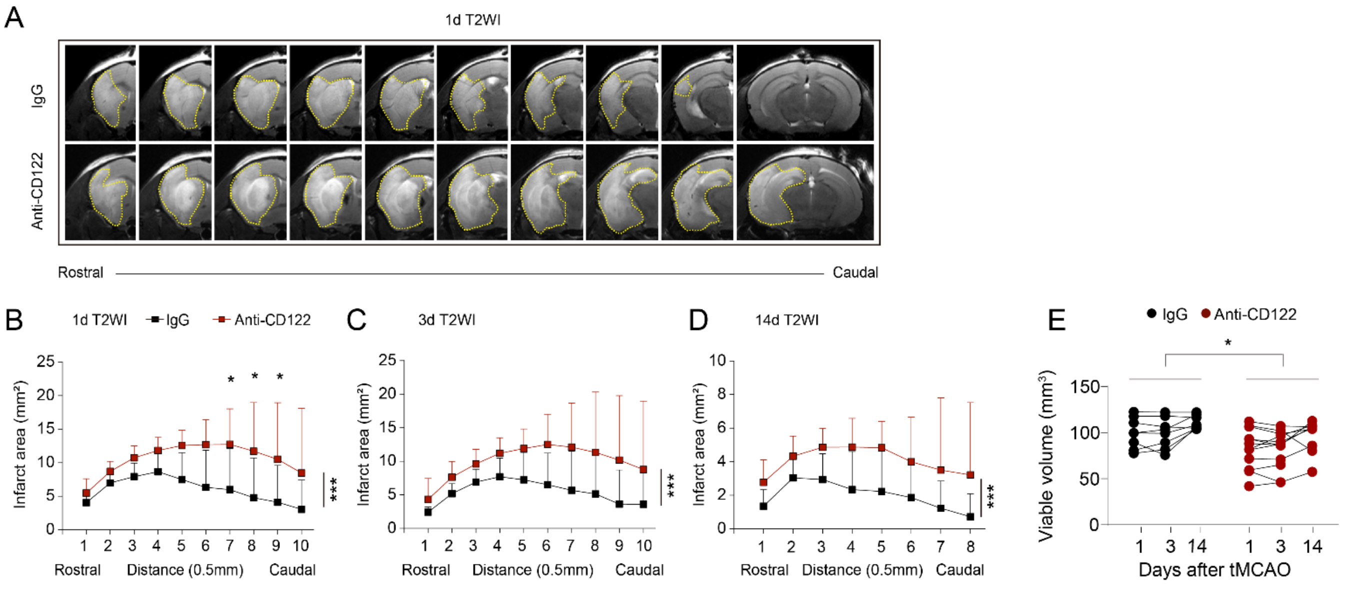

Supplemental Fig. 2. Depletion of CD8$^+$ TRLs worsens stroke outcomes. Mice were treated with CD122 mAb (100 µg) or IgG isotype control (100 µg) and subjected to tMCAO or sham operation. n=8 mice for IgG group. n=12 mice for anti-CD122 group. **(A)** Shown are rostral to caudal T2 weighted images (T2WI) taken at 1d after tMCAO in 10 coronal brain slices. *Dashed line* illustrates the boundary of brain infarct. **(B-D)** Infarct area was calculated on T2WI from 8-10 coronal sections encompassing the ischemic lesion at 1, 3, and 14d after tMCAO. Two-way ANOVA and *post hoc* Bonferroni. **(E)** Viable tissue volume was calculated on T2WI images at 1, 3, and 14d after tMCAO. Mixed-effects repeated measures ANOVA and *post hoc* Bonferroni. All data are mean±SD, *p<0.05, ***p<0.001.

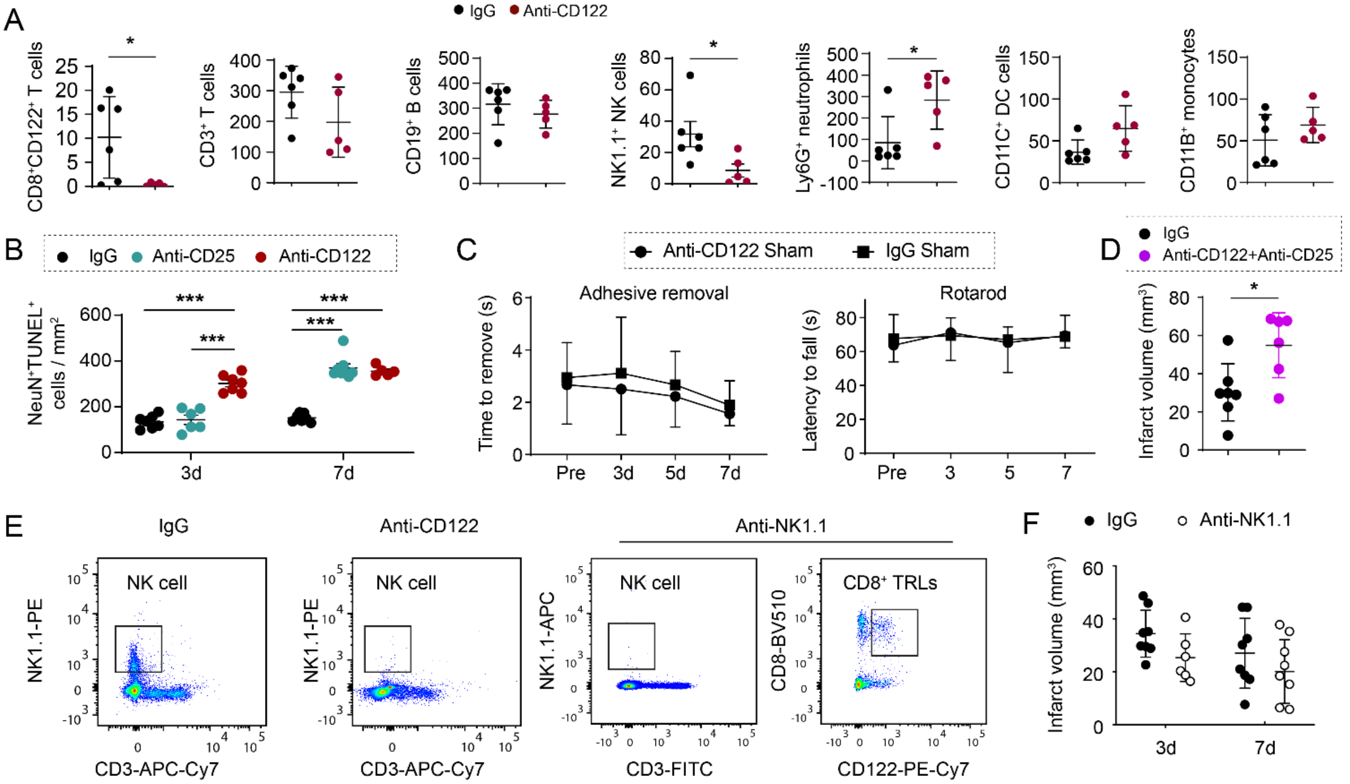

Supplemental Fig. 3. Impact of CD8$^+$CD122$^+$ TRLs on stroke outcomes. (A) Effect of anti-CD122 treatment on the number of immune cells in blood 3d after tMCAO. n=5-6/group. Two-tailed Student's t test. **(B)** Quantification of NeuN$^+$TUNEL$^+$ neurons in the peri-infarct areas 3d and 7d after tMCAO in IgG Ab (100 µg), anti-CD122 mAb (100 µg), or anti-CD25 mAb (100 µg) treated (ip, 2d before tMCAO) mice. n=5-8/group. One-way ANOVA and *post hoc* Dunnett. **(C)** Sensorimotor function was analyzed with the adhesive removal test and rotarod test in sham mice injected with anti-CD122 or isotype IgG. n=6/group. Two-way repeated measures ANOVA. **(D)** Quantification of infarct volumes in MAP2 stained sections 3d after tMCAO in anti-CD122 mAb + anti-CD25 mAb (100 µg for each mAb) treated mice or IgG treated mice. n=6-7/group. Two-tailed Student's t test. **(E-F)** Depletion of NK cells with anti-NK1.1 mAb showed no effect on infarct volume 3d after 60 min tMCAO. Mice were treated with isotype IgG (100 µg), anti-CD122 (100 µg), or anti-NK1.1 mAb (100 µg for NK cell depletion) 2d prior to 60 min tMCAO. ***(E)*** Flow cytometry plot showing NK cell depletion by anti-NK1.1 or anti-CD122 mAb 3d after stroke. ***(F)*** Infarct volume was assessed by MAP2 staining. n=6-8/group. Two-tailed Student's t test. All data are mean±SD, * $p<0.05$, *** $p<0.001$.

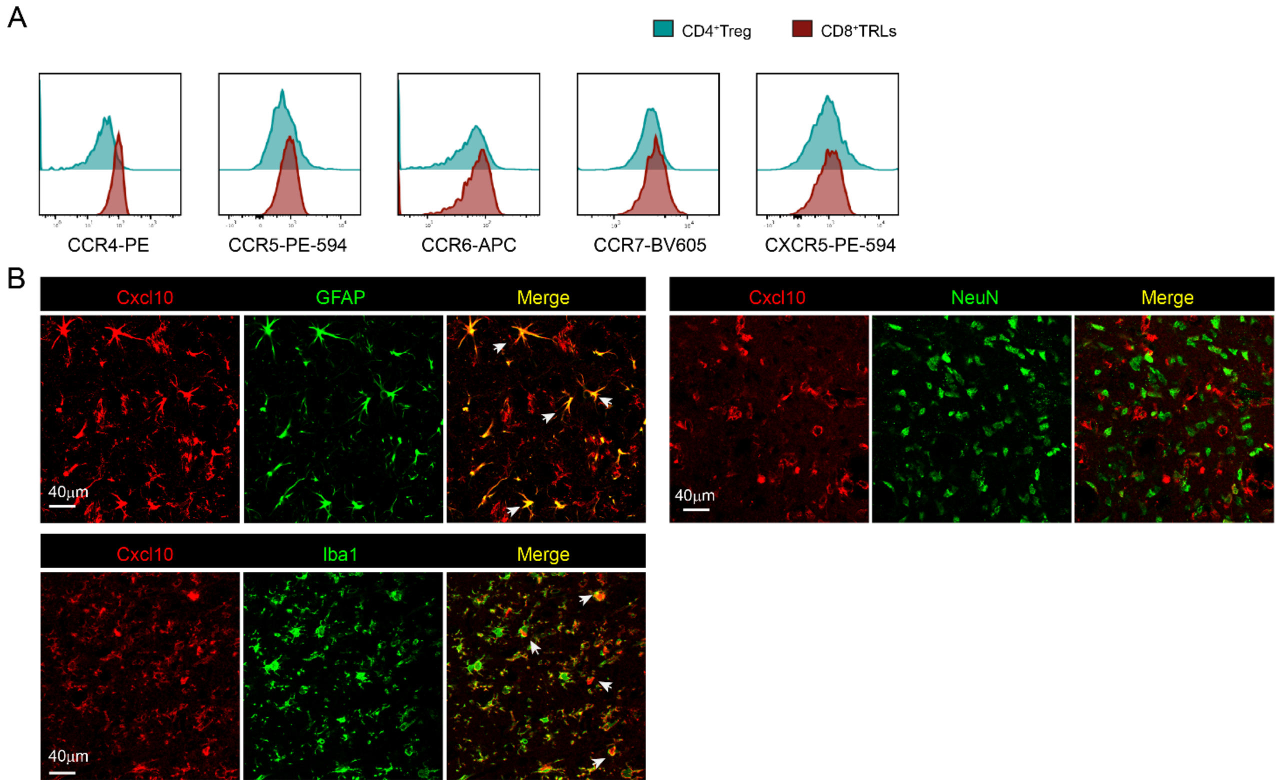

Supplemental Fig. 4. Signals for CD8$^+$ TRL recruitment and activation. Mice were subjected to 60 min tMCAO. **(A)** Flow cytometric analysis of CCR4$^+$, CCR5$^+$, CCR6$^+$, CCR7$^+$ and CXCR5$^+$ cells among CD8$^+$ TRLs and CD4$^+$ Tregs in the ischemic brain. The plots are representative from 3 animals in each group. **(B)** Expression of CXCL10 (red) in astrocytes (GFAP, green), microglia (Iba1, green) and neurons (NeuN, green) was assessed in the ischemic brain by immunostaining 3d after stroke. The arrows point to selective double-labeled cells (CXCL10/GFAP, CXCL10/Iba1).

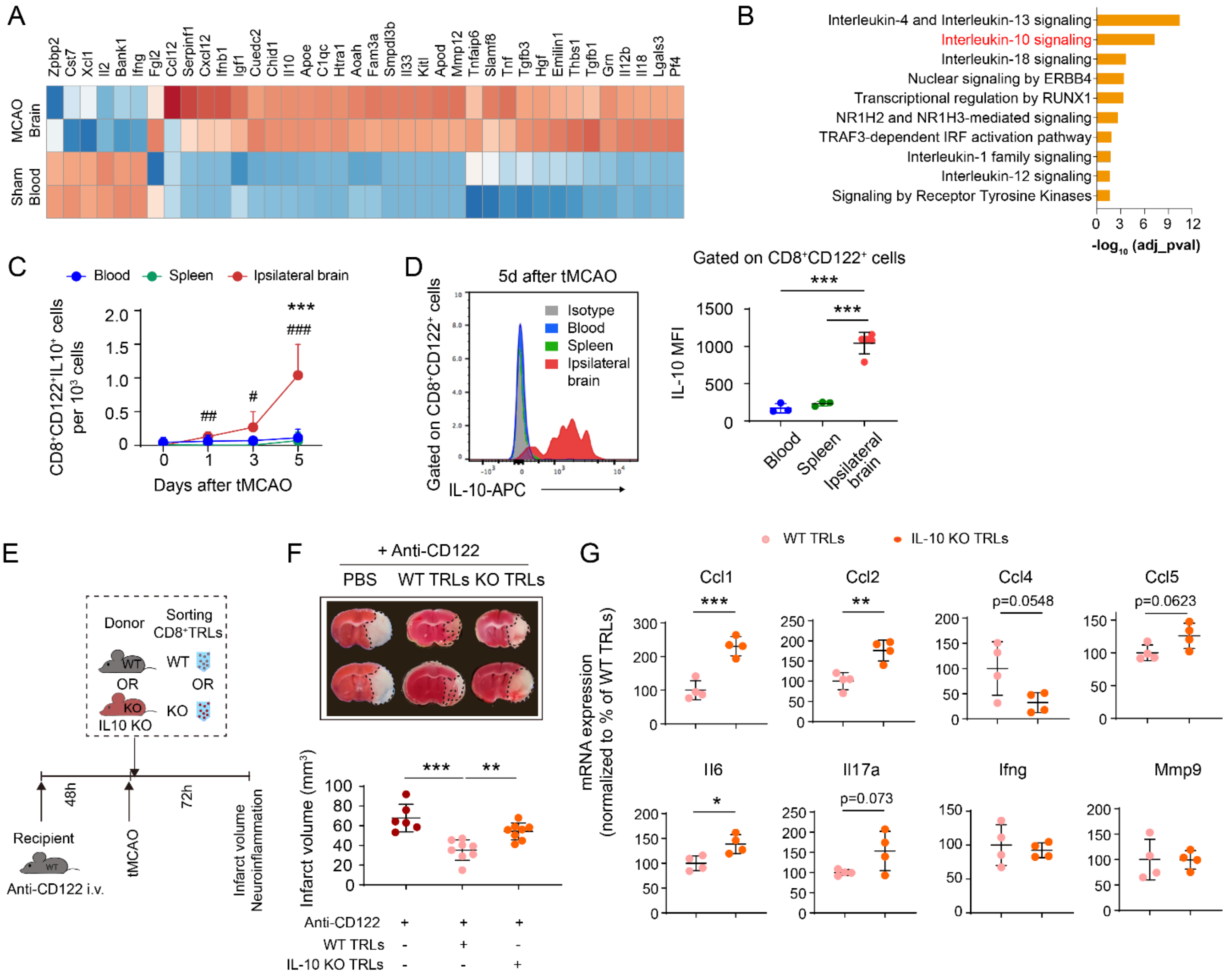

Supplemental Fig. 5. CD8$^+$ TRLs ameliorate post-stroke inflammation in an IL-10 dependent manner. (A) Heatmap showing immunoregulation-related DEGs that are higher (red) or lower (blue) in brain infiltrating TRLs from stroke mice *vs.* blood TRLs from sham mice in RNA-seq analysis. **(B)** IPA pathway analysis for signaling cascades that were activated in brain infiltrating CD8$^+$ TRLs. **(C)** The number of IL-10$^+$CD8$^+$CD122$^+$ TRLs in the blood, spleen and ipsilateral brains at indicated time points after tMCAO. n=3-8/group. ***p<0.001 *vs.* blood. #p<0.05, ##p<0.01, ###p<0.001 *vs.* spleen. One-way ANOVA and *post hoc* Bonferroni. **(D)** Quantification of the mean fluorescence intensity (MFI) of IL-10 in CD8$^+$ TRLs 5d after tMCAO. n=3-5/group. One-way ANOVA and *post hoc* Bonferroni. **(E-F)** Mice were treated with anti-CD122 mAb (100 μg) 2d prior to 60 min tMCAO. CD8$^+$ TRLs prepared from WT or IL-10 KO mice were transferred (1×10^6 cells, *i.v.*) into CD8$^+$ TRL-depleted mice 2h after stroke. Infarct volumes

were quantified by TTC staining 3d after stroke. n=6-8/group. One-way ANOVA and *post hoc* Bonferroni. **(G)** Brain inflammation was quantified by RT-PCR analysis for *Ccl1*, *Ccl2*, *Ccl4*, *Ccl5*, *Il6*, *Il17a*, *Ifng*, and *Mmp9* expression at 3d after tMCAO. n=4/group. Two-tailed Student's t test. All data are mean±SD, *p<0.05, **p<0.01, ***p<0.001.

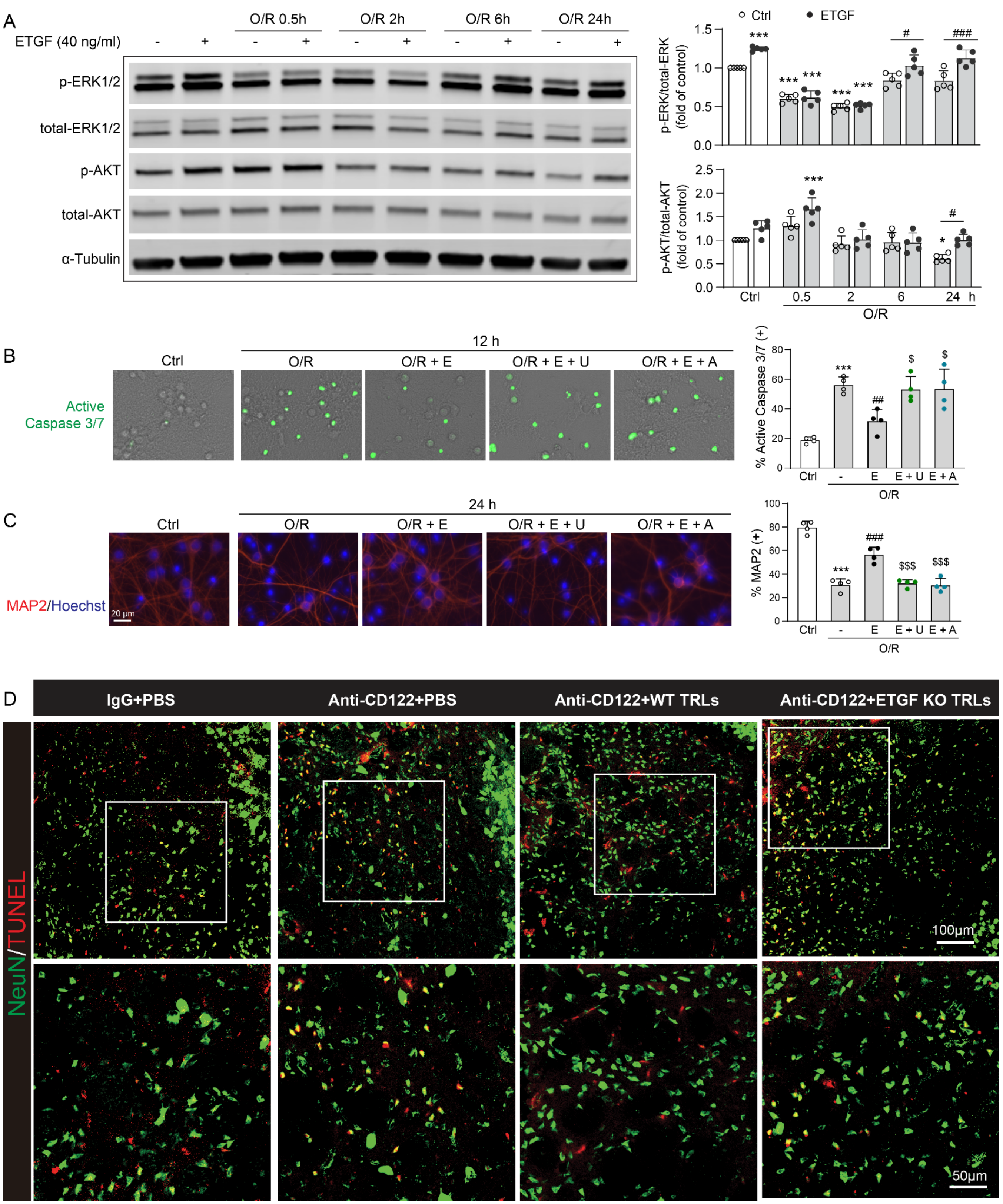

Supplemental Fig. 6. CD8^{+} TRLs ameliorate neuronal injury in an ETGF dependent manner. Primary cortical neurons were pre-treated with ETGF (E, 40 ng/ml) or PBS and subjected to OGD followed by oxygen-glucose restoration (O/R). Cells were collected at 0.5, 2, 6, 12 and 24h after O/R for Western

blotting, cell viability or cell death assays. **(A)** Western blotting for phosphorylated- or total-ERK1/2 and phosphorylated- or total-AKT were performed at the indicated time points after OGD. Data are from 5 independent experiments. **(B)** Apoptotic cell death was assessed by immunofluorescent staining for the active form of caspase-3/7 (green), and data were expressed as percentages of active caspase $3/7^+$ cells among total number of cells at 12h after O/R. While ETGF (E, 40 ng/ml) reduced apoptosis after O/R, administration of AKT inhibitor VIII (A, 5 μM) or ERK inhibitor U0126 (U, 5 μM) abolished the neuroprotective effect of ETGF. Data are from 4 independent experiments. **(C)** Neuronal survival was measured by MAP2 staining 24h after O/R. While ETGF (E, 40 ng/ml) increased cell survival after O/R, administration of AKT inhibitor VIII (A, 5 μM) or ERK inhibitor U0126 (U, 5 μM) abolished the neuroprotective effect of ETGF. Data are from 4 independent experiments. All data are mean ± SD. One way ANOVA and *post hoc* Bonferroni. ***p<0.001 vs. non-OGD control. #p<0.05, ## p<0.01, ###p<0.001 vs. OGD control or as indicated. $ p<0.05, $$$p<0.001 vs. OGD+ETGF. O/R: OGD/reperfusion. **(D)** Mice were treated with anti-CD122 mAb (100 μg) 2d prior to 60 min tMCAO. $CD8^+$ TRLs prepared from WT or ETGF KO mice were transferred (1×10^6 cells, *i.v.*) into $CD8^+$ TRL-depleted mice 2h after tMCAO. Representative images demonstrating TUNEL (red) colabeling with the neuronal marker NeuN (green) in peri-infarct areas 3d after stroke. The images in white boxes in the upper panel were enlarged in the lower panel.

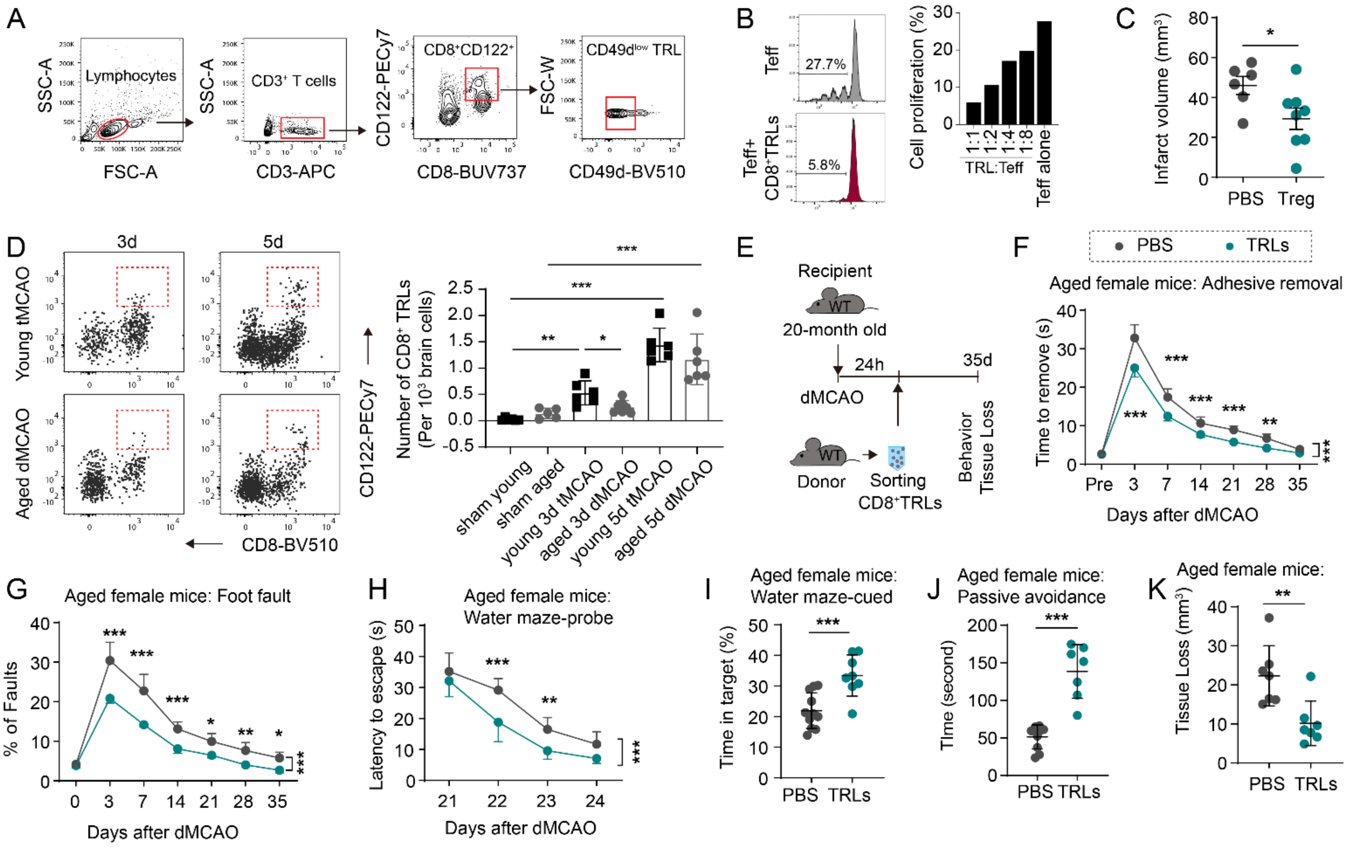

Supplemental Fig. 7. Effect of CD8⁺ TRLs on post-stroke brain injury and functional recovery. (A) CD8⁺ TRLs (CD8⁺CD122⁺CD49d^{low}) were sorted from pooled spleens of healthy young donor mice. **(B)** T effector cells (Teffs) suppression test. Teffs were labeled with CFSE (1 μM, 37°C, 10 min) and then plated at 2 × 10^5 cells per well in a U-bottom 96-well plate in the presence of CD3/CD28 activation beads to stimulate their proliferation. TRLs were added at a ratio of 1:1, 1:2, 1:4, or 1:8 to the number of Teffs, and cells were incubated for 3d. Suppression of Teff proliferation was determined by CFSE dilution on a flow cytometer. Left: Representative plots of suppression assay using CFSE-labeled Teffs incubated without or with CD8⁺ TRLs (1:1). Right: The histogram indicates Teff proliferation rate. **(C)** CD8⁺ TRLs (CD8⁺CD122⁺CD49d^{low}) were sorted from pooled spleens of healthy aged male donor mice (20-month-old). Young male stroke mice were treated intravenously with aged CD8⁺ TRLs or PBS at 2h after tMCAO. Quantification of MAP2 staining at 3d after tMCAO. n=6-8. **(D)** Comparison of the amounts of CD8⁺ TRL infiltration into the brain in young (12-week-old) and aged (20-month-old) mice after tMCAO and dMCAO, respectively. n=4-6/group. **(E-K)** Aged female (20-month-old) stroke mice were treated intravenously with 1x10^6 FACS-sorted CD8⁺ TRLs or PBS at 24h after dMCAO. n=7-11/group. ***(E)*** Experimental design for

Fig. 8J-P and Fig S7F-K. Sensorimotor dysfunction was assessed by the adhesive removal ***(F)*** and foot-fault tests ***(G)*** up to 35d after dMCAO. ***(H-I)*** Spatial learning and memory were assessed at 21d–25d after dMCAO in the Morris water maze. ***(J)*** Non-spatial memory was assessed at 35d after dMCAO using the passive avoidance test. ***(K)*** Quantification of MAP2 staining at 35d after dMCAO. *$p<0.05$, **$p<0.01$, ***$p<0.001$. Two-tailed Student's t test or Welch's t test (C, D, I, J, K), or two-way ANOVA and *post hoc* Bonferroni (F, G, H).

A Full unedited gel for Figure 5B

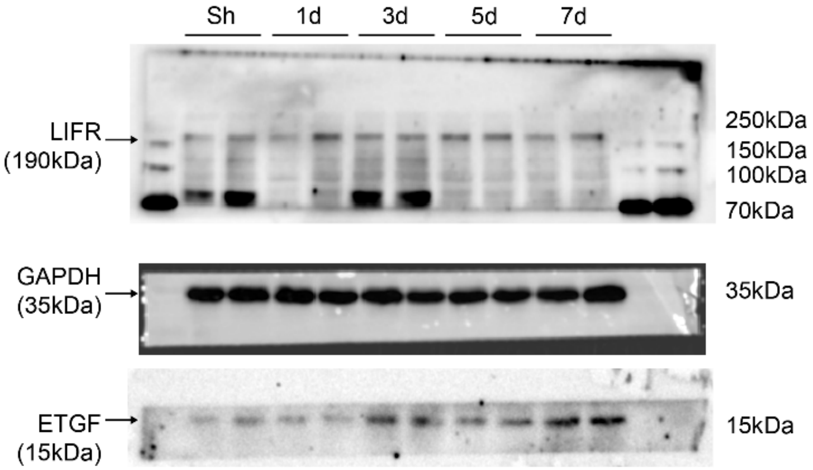

B Full unedited gel for Figure S6A

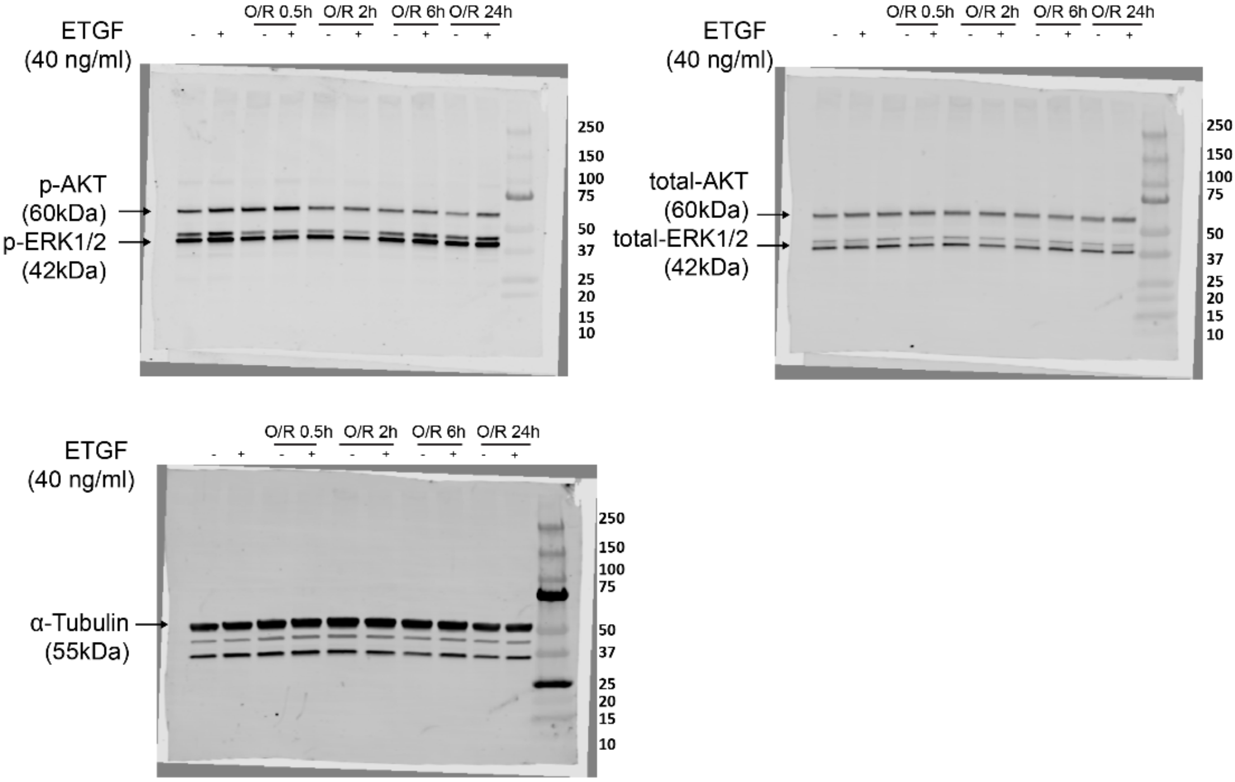

Supplemental Fig. 8. Uncut western blot gel images.

Table S4. Mortality and exclusion rates.

Group	Surgery	Treatment	Mortality	Exclusion rate
WT young	Sham	- or anti-CD122	0	0
WT young	tMCAO	- or IgG	6.04%	4.95%
WT young	tMCAO	Anti-CD122	8.86%	7.59%
WT young	tMCAO	CD122 Ab+WT TRLs	6.06%	6.06%
WT young	tMCAO	Anti-NK1.1	6.25%	6.25%
WT young	tMCAO	Anti-CD25	10%	5%
WT young	tMCAO	Anti-CD25+anti-CD122	14.29%	0
WT young	tMCAO	CD122 Ab+LIF-treated WT TRLs	0	0
WT young	tMCAO	CD122 Ab+LIF inhibitor-treated WT TRLs	0	0
WT young	tMCAO	CD122 Ab+ETGF	11.11%	11.11%
WT young	tMCAO	CD122 Ab+CXCL10 KO TRLs	0	0
WT young	tMCAO	CD122 Ab+CXCR3 KO TRLs	10%	0%
WT young	tMCAO	CD122 Ab+ETGF KO TRLs	14.29%	0%
WT young	tMCAO	CD122 Ab+IL10 KO TRLs	0	11.11%
WT young	tMCAO	Young TRLs	6%	4%
WT young	tMCAO	Aged TRLs	0%	0%
Rag1 KO	tMCAO	-	0	10%
Rag1 KO	tMCAO	Teff+TRLs	11.11%	0%
Rag1 KO	tMCAO	Teff	10%	10%

Rag1 KO	tMCAO	TRLs	0	11.11%
Aged WT male	Sham	-	0%	0%
Aged WT male	dMCAO	- or PBS	7.7%	0%
Aged WT male	dMCAO	TRLs	9%	0%
Aged female	dMCAO	PBS	9%	0%
Aged female	dMCAO	TRLs	0%	0%

Table S5. Primers for RT-PCR.

Gene	Forward primer	Reverse primer
Ccl1	AGTTCTTGGCTCCACCAGAC	CATCCTGTATCCACACGGCA
Ccl2	TGACCCCAAGAAGGAATGGG	ACCTTAGGGCAGATGCAGTT
Ccl4	CTTCTGTGCTCCAGGGTTCTC	CTGCCTCTTTTGGTCAGGAATACCA
Ccl5	AAGTGTGTGCCAACCCAGAG	CCCATTTTCCCAGGACCGAG
Cxcl9	ATG AAGTCCGCTGTTCTTTTCC	GTCTCTTATGTAGTCTTCCTT G
Cxcl10	CTAGCTCAGGCTCGTCAGTT	CCCTTGGGAAGATGGTGGTTA
Cxcl11	ATGAACAGGAAGGTCACAGC	GATGTCACATGTTTTGACGC
Il1a	AAGACAAGCCTGTGTTGCTGAAGG	TCCCAGAAGAAAATGAGGTCGGTC
Il6	TCCTACCCCAACTTCCAATGCTC	TTGGATGGTCTTGGTCCTTAGCC
Il10	CCAAGCCTTATCGGAAATGA	TTTTCACAGGGGAGAAATCG
Il17a	CCTGGACTCTCCACCGCAA	TTCCCTCCGCATTGACACAG
Mmp9	CCAGCCGACTTTTGTGGTCT	TGGCCTTTAGTGTCTGGCTG
Tnf	AGAAGTTCCCAAATGGCCTC	CCACTTGGTGGTTTGCTACG
Ifng	ATGAACGCTACACACTGCATC	CCATCCTTTTGCCAGTTCCTC
Tgfb1	TGCGCTTGCAGAGATTAAAA	CGTCAAAAGACAGCCACTCA
Tgfa	CTTCTTGGTGCAAAGGCTCG	TCGACTGACGAATGGGCTTG
Gapdh	CCCTTAAGAGGGATGCTGCC	TACGGCCAAATCCGTTCACA

Table S6. Statistical Analysis

FIGURE 1	n	DATA STRUCTURE	TEST USED	STATISTIC	P VALUE
1A	Day0: n=4,<br>Day1: n=8,<br>Day3: n=5,<br>Day5: n=5,<br>Day7: n=6,<br>Day14: n=7,<br>Identical number for both groups at all time points.	Normal distribution	Unpaired t test	t(day0)=0.000,<br>t(day1)=3.953,<br>t(day3)=15.32,<br>t(day5)=9.161,<br>t(day7)=2.4,<br>t(day14)=9.133	p(day0)>0.9999,<br>p(day1)=0.0014,<br>p(day3)<0.0001,<br>p(day5)=0.0007,<br>p(day7)=0.0394,<br>p(day14)<0.0001
1B	CD103: n=4,<br>IL-10: n=4,<br>HELIOS: n=4,<br>Identical number for both groups.	Normal distribution	Unpaired t test	t(CD103)=3.700,<br>t(IL-10)=6.216,<br>t(HELIOS)=10.21	p(CD103)=0.0101,<br>p(IL-10)=0.0078,<br>p(HELIOS)<0.0001
1E	IgG: n=8,<br>Anti-CD122: n=12	Normal distribution	2-way repeated ANOVA, Bonferroni post hoc	F(1,18)=4.495	p=0.0481,<br>p(baseline)=/,<br>p(ischemia)>0.9999,<br>p(reperfusion)=0.2743
1G	IgG: n=8,<br>Anti-CD122:<br>n(day1)=12;<br>n(day3)=12;<br>n(day14)=9	Normal distribution	Mixed-effects repeated measurement, Bonferroni post hoc	F(1,18)=6.265	p=0.0222,<br>p(day1)=0.0473,<br>p(day3)=0.0642,<br>p(day14)=0.0281
1H	IgG: n=8,<br>Anti-CD122: n=12	Normal distribution	2-way repeated ANOVA, Bonferroni post hoc	F(1,18)=10.62	p=0.0044,<br>p(day1)=0.0090,<br>p(day3)=0.0047
1J	IgG+PBS: n=6,<br>Anti-CD122+PBS: n=6,<br>Anti-CD122+CD8$^+$ TRL: n=6	Normal distribution	1-way ANOVA, Dunnett post hoc	F(2,15)=11.21	p=0.0011,<br>p(IgG+PBS vs Anti-CD122+PBS)=0.0013,<br>p(Anti-CD122+PBS vs Anti-CD122+CD8$^+$ TRL)=0.0027
1K	Day3: n(IgG)=7,<br>n(anti-CD25)=7,<br>n(anti-CD122)=8;<br>Day7: n(IgG)=8,<br>n(anti-CD25)=7,<br>n(anti-CD122)=8	Normal distribution	1-way ANOVA, Dunnett post hoc	Day 3 F(2,19)=5.317,<br>Day 7, F(2, 20)=6.403	Day3:<br>p=0.0147,<br>p(IgG vs anti-CD25)=0.4843,<br>p(IgG vs anti-CD122)=0.0091;<br>Day7:<br>p=0.0071<br>p(IgG vs anti-CD25)=0,0130,<br>p(IgG vs anti-CD122)=0.0096
1L	IgG: n=10,<br>Anti-CD25: n=9,<br>Anti-CD122: n=8	Normal distribution	2-way repeated ANOVA, Dunnett post hoc	F(2,24)=16.47	p<0.0001;<br>Pre: p(IgG vs anti-CD25)=0.8867,<br>p(IgG vs anti-CD122)=0.7962;<br>day3: p(IgG vs anti-CD25)=0.4484,<br>p(IgG vs anti-CD122)<0.0001;<br>day5: p(IgG vs anti-CD25)=0.0001,<br>p(IgG vs anti-CD122)<0.0001;<br>day7: p(IgG vs anti-CD25)<0.0001,<br>p(IgG vs anti-CD122)<0.0001
1M	IgG: n=10,<br>Anti-CD25: n=10,<br>Anti-CD122: n=8	Normal distribution	2-way repeated ANOVA, Dunnett post hoc	F(2,25)=9.829	p=0.0007;<br>Pre: p(IgG vs anti-CD25)=0.9713,<br>p(IgG vs anti-CD122)=0.9986;<br>day3: p(IgG vs anti-CD25)=0.5733,<br>p(IgG vs anti-CD122)<0.0001;<br>day5: p(IgG vs anti-CD25)=0.0424,<br>p(IgG vs anti-CD122)<0.0001;<br>day7: p(IgG vs anti-CD25)=0.0053,<br>p(IgG vs anti-CD122)=0.0167
1N	IgG: n=10,<br>Anti-CD25: n=9,<br>Anti-CD122: n=8	Normal distribution	2-way repeated ANOVA, Dunnett post hoc	F(2,24)=7.172	p=0.0036;<br>Pre: p(IgG vs anti-CD25)=0.8974,<br>p(IgG vs anti-CD122)=0.9961;<br>day3: p(IgG vs anti-CD25)=0.9995,<br>p(IgG vs anti-CD122)=0.0328;<br>day5: p(IgG vs anti-CD25)=0.0006,<br>p(IgG vs anti-CD122)=0.0015;

					day7: p(IgG vs anti-CD25)=0.0017, p(IgG vs anti-CD122)=0.3357
**FIGURE 2**	**n**	**DATA STRUCTURE**	**TEST USED**	**STATISTIC**	**P VALUE**
2D	*$CD4^+$ Treg: n=3, $CD8^+$ TRL: n=3*	*Normal distribution*	*Unpaired t test*	*t=15.48*	*p=0.0001*
**FIGURE 3**	**n**	**DATA STRUCTURE**	**TEST USED**	**STATISTIC**	**P VALUE**
3A	*1d: sham=3, Cxcl9=3, Cxcl10=3, Cxcl11=3; 3d: sham=3, Cxcl9=3, Cxcl10=3, Cxcl11=3*	*Normal distribution*	*Unpaired t test*	*1d: t(CXCL9)=4.370, t(CXCL10)=2.735, t(CXCL11)=2.067; 3d: t(CXCL9)=4.158, t(CXCL10)=3.545, t(CXCL11)=5.532*	*1d: p(CXCL9)=0.012, p(CXCL10)=0.0522, p(CXCL11)=0.1076 ; 3d: p(CXCL9)=0.0142, p(CXCL10)=0.0239, p(CXCL11)=0.0052*
3B	*Blood: n(sham)=6, n(tMCAO)=6; Brain: n(sham)=6, n(tMCAO)=5*	*Normal distribution*	*Unpaired t test*	*Blood: t=1.431; Brain: t=2.691*	*p(blood)=0.1828; p(brain)=0.0248;*
3E	*n=6 for both groups*	*Normal distribution*	*Unpaired t test*	*t=5.980*	*P=0.0001*
3F	*WT: n=3; Cxcr3 KO: n=3*	*Normal distribution*	*Unpaired t test*	*t=3.228*	*p=0.0321*
3H	*IgG+PBS: n=8; Anti-CD122: n(PBS)=8, n(WT TRL)=6, n(Cxcr3 KO TRL)=6*	*Normal distribution*	*1-way ANOVA, Bonferroni post hoc*	*F (3,24)=13.62*	*p<0.0001; p(IgG+PBS vs Anti-CD122+PBS)<0.0001, p(Anti-CD122+PBS vs WT TRL)=0.0006, p(IgG+PBS vs Anti- CD122+WT TRL)>0.9999, p(IgG+PBS vs Anti-CD122+CXCR3 KO TRL)=0.0045, p(Anti-CD122+WT TRL vs Anti-CD122+CXCR3 KO TRL)=0.024, p(Anti-CD122+PBS vs Anti-CD122+CXCR3 KO TRL)>0.9999*
3I	*TNFα: n(ctrl)=3, n(Teff)=3, n(Teff+WT)=3, n(Teff+CXCR3 KO)=3; IL-4: n(ctrl)=3, n(Teff)=3, n(Teff+WT)=3, n(Teff+CXCR3 KO)=3*	*Normal distribution*	*1-way ANOVA, Bonferroni post hoc*	*TNFα: F(3,8)=974, IL-4:F(3,8)=313*	*p(TNFα)<0.0001, p(Teff vs Teff+WT)<0.0001, p(Teff vs Teff+CXCR3 KO)=0.0002, p(Teff+WT vs Teff+CXCR3 KO)=0.0592; p(IL-4)<0.0001, p(Teff vs Teff+WT)=0.0164, p(Teff vs Teff+CXCR3 KO)=0.0044, p(Teff+WT vs Teff+CXCR3 KO)>0.9999;*
**FIGURE 4**	**n**	**DATA STRUCTURE**	**TEST USED**	**STATISTIC**	**P VALUE**
4D(Il1a)	*1d: sham=7, IgG=5, anti-CD122=3; 3d: sham= 3, IgG=3, anti-CD122=3*	*Normal distribution*	*2-way ANOVA, Bonferroni post hoc*	*F(2,18)=22.96*	*p<0.0001; 1d: p(sham vs IgG)=0.0005, p(sham vs anti-CD122)=0.0009, p(IgG vs anti-CD122)>0.9999; 3d: p(sham vs IgG)>0.9999, p(sham vs anti-CD122)=0.0002, p(IgG vs anti-CD122)=0.0007*
4D(Tnf)	*1d: sham=7, IgG=5, anti-CD122=3; 3d: sham= 3, IgG=3, anti-CD122=3*	*Normal distribution*	*2-way ANOVA, Bonferroni post hoc*	*F(2,18)=18.93*	*p<0.0001; 1d: p(sham vs IgG)=0.0033, p(sham vs anti-CD122)=0.0056, p(IgG vs anti-CD122)>0.9999; 3d: p(sham vs IgG)=0.2952, p(sham vs anti-CD122)=0.0004, p(IgG vs anti-CD122)=0.0183*
4D(Ifng)	*1d: sham=7, IgG=4, anti-CD122=3; 3d: sham= 3, IgG=3, anti-CD122=3*	*Normal distribution*	*2-way ANOVA, Bonferroni post hoc*	*F(2,17)=19.30*	*p<0.0001; 1d: p(sham vs IgG)=0.6568, p(sham vs anti-CD122)=0.2584, p(IgG vs anti-CD122)>0.9999; 3d: p(sham vs IgG)>0.9999, p(sham vs anti-CD122)<0.0001, p(IgG vs anti-CD122)<0.0001*
4D(Il6)	*1d: sham=7, IgG=5, anti-CD122=3;*	*Normal distribution*	*2-way ANOVA, Bonferroni post hoc*	*F(2,18)=8.730*	*P=0.0022;*

	3d: sham= 3, IgG=3, anti-CD122=3				*1d: p(sham vs IgG)=0.1745, p(sham vs anti-CD122)=0.0694, p(IgG vs anti-CD122)>0.9999; 3d: p(sham vs IgG)>0.9999, p(sham vs anti-CD122)=0.0102, p(IgG vs anti-CD122)=0.0646*
4D(Il10)	*1d: sham=7, IgG=4, anti-CD122=3; 3d: sham= 3, IgG=3, anti-CD122=3*	*Normal distribution*	*2-way ANOVA, Bonferroni post hoc*	*F(2,17)=1.691*	*p=0.2139; 1d: p(sham vs IgG)=0.7743, p(sham vs anti-CD122)=0.0521, p(IgG vs anti-CD122)=0.5208; 3d: p(sham vs IgG)>0.9999, p(sham vs anti-CD122)>0.9999, p(IgG vs anti-CD122)>0.9999*
4D(Tgfb1)	*1d: sham=7, IgG=4, anti-CD122=3; 3d: sham= 3, IgG=3, anti-CD122=3*	*Normal distribution*	*2-way ANOVA, Bonferroni post hoc*	*F(2,17)=9.128*	*p=0.0020; 1d: p(sham vs IgG)=0.0019, p(sham vs anti-CD122)=0.0222, p(IgG vs anti-CD122)>0.9999; 3d: p(sham vs IgG)=0.4335, p(sham vs anti-CD122)=0.1945, p(IgG vs anti-CD122)>0.9999*
4F	*n=3 for both groups at each cell type*	*Normal distribution*	*Unpaired t test*	*t(neutrophil)=1.967 ; t(DC)=1.155 ; t(macrophage)=1.716 ; t(T cell)=1.138 ; t(B cell)=1.709*	*p(neutrophil)=0.1206 ; p(DC)=0.3125 ; p(macrophage)=0.1614 ; p(T cell)=0.3187 ; p(B cell)=0.1627*
4G	*naïve=9; Teff=8; Teff+TRL=8; TRL=8*	*Normal distribution*	*1-way ANOVA, Bonferroni post hoc*	*F (3,29)=9.948*	*p=0.0001; p(naïve vs Teff)=0.0202, p(naive vs TRL)=0.0404, p(Teff vs Teff+TRL)=0.0054*
**FIGURE 5**	**n**	**DATA STRUCTURE**	**TEST USED**	**STATISTIC**	**P VALUE**
5B	*LIFR: n=4 ETGF: n=4*	*Normal distribution*	*1-way ANOVA, Dunnett post hoc*	*F(LIFR)=2.865; F(ETGF)=6.919*	*p(LIFR)=0.0602, p(1d vs sham)=0.1376, p(3d vs sham)=0.0467, p(5d vs sham)=0.0373, p(7d vs sham)=0.5236; p(ETGF)=0.0023, p(1d vs sham)=0.9987, p(3d vs sham)=0.0065, p(5d vs sham)=0.0215, p(7d vs sham)=0.0469*
5C	*Blood: sham=3, tMCAO=3; Spleen: sham=3, tMCAO=4; Brain=3*	*Normal distribution*	*1-way ANOVA, Dunnett post hoc*	*F(4,11)=124.9*	*P<0.0001; P<0.0001 for Brain tMCAO vs all other groups.*
5E	*n=6 for all groups*	*Normal distribution*	*1-way ANOVA, Bonferroni post hoc*	*F(3,20)=16.13*	*p<0.0001; p(blood sham vs blood tMCAO)>0.9999; p(brain sham vs brain tMCAO)<0.0001*
**FIGURE 6**	**n**	**DATA STRUCTURE**	**TEST USED**	**STATISTIC**	**P VALUE**
6A(Lifr)	*n(ctrl)=12; n(Cl)=6; n(IP)=7*	*Non normal distribution*	*Kruskal-Wallis test, Dunn's post hoc*	*Kruskal-Wallis statistic=19.69*	*p<0.0001; p(ctrl vs Cl)>0.9999, p(ctrl vs IP)<0.0001, p(Cl vs IP)=0.0011*
6A(Etgf)	*n(ctrl)=16; n(Cl)=8; n(IP)=16*	*Non normal distribution*	*Kruskal-Wallis test, Dunn's post hoc*	*Kruskal-Wallis statistic=29.46*	*p<0.0001; p(ctrl vs Cl)=0.2702, p(ctrl vs IP)=0.0002, p(Cl vs IP)<0.0001*
6A(Il10)	*n(ctrl)=6; n(Cl)=6; n(IP)=12*	*Non normal distribution*	*Kruskal-Wallis test, Dunn's post hoc*	*Kruskal-Wallis statistic=16.13*	*p=0.0003; p(ctrl vs Cl)>0.9999, p(ctrl vs IP)=0.0123, p(Cl vs IP)=0.0009*
6B	*n(CL)=6; n(IP)=6; n(LIFab)=4*	*Normal distribution*	*1-way ANOVA, Bonferroni post hoc*	*ETGF: F(2,13)=8.904; IL10: F(2,13)= 4.550*	*ETGF: p=0.0037; p(CL vs IL)=0.0051; p(CL vs LIFab)>0.9999; p(IP vs LIFab)=0.0241 IL10:*

					p=0.0318; p(CL vs IP)=0.0446; p(CL vs LIFab)>0.9999; p(IP vs LIFab)=0.1279
6E	n=3 for both groups	Normal distribution	Unpaired t test;	t(ETGF MFI)=5.383; t(IL10 MFI)=3.472;	p(ETGF MFI)=0.0058; p(IL10 MFI)=0.0255;
6F	n(PBS)=5; n(non-treated $CD8^+$ TRLs)=6; n(LIF-treated $CD8^+$ TRLs)=6; n(LIFR inhibitor-treated $CD8^+$ TRLs)=6	Normal distribution	1-way ANOVA, Bonferroni post hoc	F(3,19)=15.68	p<0.0001; p(PBS vs non-treated treated $CD8^+$ TRLs)=0.0088; p(PBS vs LIF-treated treated $CD8^+$ TRLs)<0.0001; p(PBS vs LIFR inhibitor-treated treated $CD8^+$ TRLs)=0.6027; p(non-treated $CD8^+$ TRLs vs LIF-treated $CD8^+$ TRLs)=0.0452 p(LIF-treated $CD8^+$ TRLs vs LIFR inhibitor-treated treated $CD8^+$ TRLs)=0.0004
6G	For both 1d and 3d: n(PBS)=5; n(non-treated $CD8^+$ TRLs)=6; n(LIF-treated $CD8^+$ TRLs)=6; n(LIFR inhibitor-treated $CD8^+$ TRLs)=6	Normal distribution	1-way ANOVA, Bonferroni post hoc	1d: F(3,19)=11.16; 3d: F(3,19)=8.546	1d: p=0.0002; p(PBS vs non-treated treated $CD8^+$ TRLs)=0.0508; p(PBS vs LIF-treated treated $CD8^+$ TRLs)=0.0002; p(PBS vs LIFR inhibitor-treated treated $CD8^+$ TRLs)>0.9999; p(non-treated $CD8^+$ TRLs vs LIF-treated $CD8^+$ TRLs)=0.0924; p(LIF-treated $CD8^+$ TRLs vs LIFR inhibitor-treated treated $CD8^+$ TRLs)=0.0014 3d: p=0.0008; p(PBS vs non-treated treated $CD8^+$ TRLs)=0.1495; p(PBS vs LIF-treated treated $CD8^+$ TRLs)=0.0011; p(PBS vs LIFR inhibitor-treated treated $CD8^+$ TRLs)=0.9681; p(non-treated $CD8^+$ TRLs vs LIF-treated $CD8^+$ TRLs)=0.1417 p(LIF-treated $CD8^+$ TRLs vs LIFR inhibitor-treated treated $CD8^+$ TRLs)=0.0034
**FIGURE 7**	**n**	**DATA STRUCTURE**	**TEST USED**	**STATISTIC**	**P VALUE**
7C	n=4 for both groups	Normal distribution	unpaired t test	t(%ETGF)=4.683; t(ETGF MFI)=2.982	p(%ETGF)=0.0034; p(ETGF MFI)=0.0246
7F	control=5, OGD=7, OGD WT TRL CM=5, OGD KO TRL CM=7	Normal distribution	1-way ANOVA, Bonferroni post hoc	F(3,20)=38.72	p<0.0001; p(control vs OGD)<0.0001, p(control vs OGD WT TRL CM)=0.0001, p(control vs OGD KO TRL CM)<0.0001, p(OGD vs OGD WT TRL CM)=0.0075, p(OGD vs OGD KO TRL CM)>0.9999, p(OGD WT TRL CM vs OGD KO TRL CM)=0.0223
7G	N=6-7	Normal distribution	1-way ANOVA, Bonferroni post hoc	F(9,55)=56.63	p<0.0001; p(OGD vs ctrl)<0.0001, p(OGD vs OGD ETGF)<0.001, p(OGD ETGF vs OGD ETGF U2.5)=0.0085, p(OGD ETGF vs OGD ETGF U5)<0.0001, p(OGD ETGF vs OGD ETGF A2.5)=0.0139, p(OGD ETGF vs OGD ETGF A5)<0.0001,

7H	*IgG=8,<br>Anti-CD122=6,<br>ETGF KO TRLs=6*	*Normal distribution*	*1-way ANOVA,<br>Bonferroni post hoc*	*F(2,17)=3.816*	*p=0.0428;<br>p(IgG vs Anti-Cd122)=0.0404,<br>p(IgG vs KO TRL)=0.6034,<br>p(Anti-Cd122 vs KO TRL)=0.5979*
7I	*WT=5, ETGF=7,*	*Normal distribution*	*Unpaired t test*	*t=2.357*	*p=0.0402*
7K	*n(IgG)=4, n(anti-CD122)=4, n(anti-CD122+WT)=6, n(anti-CD122+KO)=4*	*Normal distribution*	*1-way ANOVA,<br>Bonferroni post hoc*	*cell density:<br>F(3,14)=55.13;<br>Tunel$^+$/NeuN$^+$%:<br>F(3,14)=130.5*	*Tunel$^+$/NeuN$^+$ density:<br>p<0.0001,<br>p(IgG vs anti-CD122)<0.0001,<br>p(IgG vs CD122+WT)<0.9999,<br>p(IgG vs anti-CD122+KO)<0.0001,<br>p(anti-CD122 vs anti-CD122+WT)<0.0001,<br>p(anti-CD122 vs anti-CD122+KO)>0.9999,<br>p(anti-CD122+WT vs anti-CD122+KO)<0.0001;<br>Tunel$^+$/NeuN$^+$%:<br>p<0.0001;<br>p(IgG vs anti-CD122)<0.0001,<br>p(IgG vs CD122+WT)<0.9999,<br>p(IgG vs anti-CD122+KO)<0.0001,<br>p(anti-CD122 vs anti-CD122+WT)<0.0001,<br>p(anti-CD122 vs anti-CD122+KO)>0.9999,<br>p(anti-CD122+WT vs anti-CD122+KO)<0.0001*

**FIGURE 8**	**n**	**DATA STRUCTURE**	**TEST USED**	**STATISTIC**	**P VALUE**
8C	*PBS=6, TRL0.5=8, TRL1=7, TRL2=6*	*Normal distribution*	*1-way ANOVA,<br>Dunnett post hoc*	*F(3,23)=2.577*	*p=0.0784,<br>p(PBS vs TRLs 1)=0.0482,<br>p(PBS vs TRLs 0.5)=0.1269,<br>p(PBS vs TRLs 2)=0.7370*
8D	*PBS=6, TRL=7*	*Non normal distribution*	*Mann-Whitney test*	*U(0d)=10,<br>U(1d)=2.5,<br>U(2d)=6,<br>U(3d)=13*	*p(0d)=0.1026;<br>p(1d)=0.0035,<br>p(2d)=0.0326,<br>p(3d)=0.2861*
8E	*PBS=8, TRL=12*	*Normal distribution*	*2-way repeated ANOVA, Bonferroni post hoc*	*F(1,18)=21.43*	*p=0.0002;<br>p(pre)>0.9999,<br>p(3d)=0.0067,<br>p(5d)=0.0156,<br>p(7d)=0.0208,<br>p(10d)=0.0010,<br>p(14d)=0.0185*
8F	*PBS=8, TRL=12*	*Normal distribution*	*2-way repeated ANOVA, Bonferroni post hoc*	*F(1,18)=8.364*	*p=0.0097;<br>p(pre)>0.9999,<br>p(3d)=0.1572,<br>p(5d)=0.0716,<br>p(7d)=0.2492,<br>p(10d)=0.0349,<br>p(14d)=0.4164*
8H	*PBS=8, TRL=12*	*Normal distribution*	*2-way repeated ANOVA, Bonferroni post hoc*	*F(1,18)=5.497*	*p=0.0307;<br>p(pre)>0.9999,<br>p(10d)=0.7436,<br>p(11d)=0.2756,<br>p(12d)=0.0350,<br>p(13d)=0.0264*
8I	*PBS=8, TRL=12*	*Normal distribution*	*Unpaired t test*	*t=1.090*	*p=0.2901*
8J	*PBS=10, TRLs=10*	*Normal distribution*	*2-way repeated ANOVA, Bonferroni post hoc*	*F(1,18)=28.93*	*p<0.0001,<br>p(pre)>0.9999,<br>p(3d)<0.0001,<br>p(7d)<0.0001,<br>p(14d)=0.1029,<br>p(21d)=0.0041,<br>p(28d)<0.0002,<br>p(35d)=0.0003*

8K	*PBS=10, TRLs=10*	*Normal distribution*	*2-way repeated ANOVA, Bonferroni post hoc*	*F(1,18)=102.2*	*p<0.0001, p(pre)>0.9999, p(3d)<0.0001, p(7d)<0.0001, p(14d)<0.0001, p(21d)<0.0001, p(28d)=0.0244, p(35d)=0.2560*
8L	*PBS=10, TRLs=10*	*Normal distribution*	*2-way repeated ANOVA, Bonferroni post hoc*	*F(1,18)=115.6*	*p<0.0001, p(pre)>0.9999, p(3d)<0.0001, p(7d)<0.0001, p(14d)<0.0001, p(21d)<0.0001, p(28d)<0.0001, p(35d)<0.0001*
8M	*PBS=10, TRLs=10*	*Normal distribution*	*2-way repeated ANOVA, Bonferroni post hoc*	*F(1,18)=29.21*	*p<0.0001, p(21d)=0.0602, p(22d)<0.0001, p(23d)=0.0350, p(24d)=0.3231*
8N	*PBS=10, TRLs=10*	*Normal distribution*	*Unpaired t test*	*t=4.295*	*P=0.0004*
8O	*PBS=10, TRLs=10*	*Normal distribution*	*Unpaired t test*	*t=4.971*	*p<0.0001*
8P	*PBS=10, TRLs=10*	*Normal distribution*	*Unpaired t test*	*t=2.955*	*P=0.0085*
**FIGURE S1**	**n**	**DATA STRUCTURE**	**TEST USED**	**STATISTIC**	**P VALUE**
S1 B	*d0: CD8=4,CD4=5; d3: CD8=5, CD4=4; d5: CD8=5, CD4=5*	*Normal distribution*	*2-way ANOVA, Fisher's LSD*	*F(1,22)=12.53*	*P(group)=0.0018; D0: p(CD4 vs CD8)=0.9342 d3: p(CD4 vs CD8)=0.0004 d5: p(CD4 vs CD8)=0.0826*
S1C	*n=4*	*Normal distribution*	*1-way ANOVA, Bonferroni post hoc*	*F(4,15)=72.32*	*P<0.0001, p(ctrl vs 5d core)<0.0001, p(ctrl vs 5d peri) <0.0001, p(5d core vs CD122Ab core)<0.0001 p(5d peri-infarct vs CD122Ab peri-infarct)<0.0001*
S1 E blood	*$CD8^+$TRLs: d0=3, d1=8, d3=7, d5=5, d7=6, d14=4; $CD4^+$ Treg: d0=3, d1=5, d3=5, d5=4, d7=4, d14=5*	*Normal distribution*	*1-way ANOVA, Dunnett post hoc*	*F($CD8^+$ TRLs)=15.20; F($CD4^+$ Treg)=73.65*	*p($CD8^+$ TRLs)<0.0001; p(d0 vs d1)<0.0001; p(d0 vs d3)=0.0019; p(d0 vs d5)=0.9926; p(d0 vs d7)=0.9910; p(d0 vs d14)=0.3897. p($CD4^+$ Treg)<0.0001; p(d0 vs d1)<0.0001; p(d0 vs d3)<0.0001; p(d0 vs d5)<0.0001; p(d0 vs d7)=0.0013; p(d0 vs d14)=0.4580.*
S1 E spleen	*$CD8^+$ TRLs: d0=5, d1=6, d3=6, d5=5, d7=6, d14=7; $CD4^+$ Treg: d0=3, d1=3, d3=6, d5=4, d7=3, d14=5*	*Normal distribution*	*1-way ANOVA, Dunnett post hoc*	*F($CD8^+$ TRLs)=13.05; F($CD4^+$ Treg)=14.78*	*p($CD8^+$ TRLs)<0.0001; p(d0 vs d1)<0.0001; p(d0 vs d3)=0.0346; p(d0 vs d5)=0.6306; p(d0 vs d7)=0.0265; p(d0 vs d14)=0.9684; p($CD4^+$ Treg)<0.0001; p(d0 vs d1)=0.8671; p(d0 vs d3)=0.0002; p(d0 vs d5)<0.0001; p(d0 vs d7)=0.0001; p(d0 vs d14)=0.0002*
**FIGURE S2**	**n**	**DATA STRUCTURE**	**TEST USED**	**STATISTIC**	**P VALUE**
S2 B	*IgG=8; Anti-CD122=12*	*Normal distribution*	*2-way ANOVA, Bonferroni post hoc*	*F(1,180)=41.87*	*p<0.0001; p(1)>0.9999; p(2)>0.9999; p(3)>0.9999; p(4)>0.9999; p(5)=0.2560; p(6)=0.0518; p(7)0.0308; p(8)=0.0238; p(9)=0.0499; p(10)=0.1712*
S2 C	*IgG=8; Anti-CD122=12*	*Normal distribution*	*2-way ANOVA, Bonferroni post hoc*	*F(1,180)=33.71*	*p<0.0001; p(1)>0.9999; p(2)>0.9999; p(3)>0.9999; p(4)>0.9999; p(5)=0.6246; p(6)=0.1683;*

					p(7)=0.1035; p(8)=0.1371;<br>p(9)=0.0887; p(10)=0.3808
S2 D	IgG=8; Anti-CD122=9	Normal distribution	2-way ANOVA,<br>Bonferroni post hoc	F(1,120)=28.48	p<0.0001;<br>p(1)>0.9999; p(2)>0.9999;<br>p(3)=0.6710; p(4)=0.1894;<br>p(5)=0.1588; p(6)=0.4506;<br>p(7)=0.3311; p(8)=0.2091
S2 E	IgG: d1=8, d3=8,<br>d14=8;<br>Anti-CD122: d1=12,<br>d3=12, d14=9	Normal distribution	Mixed-effects<br>analysis, Bonferroni<br>post hoc	F(1,18)=5.734	p=0.0277;<br>p(d1)=0.1611, p(d3)=0.1244,<br>p(d14)=0.1132
**FIGURE S3**	**n**	**DATA STRUCTURE**	**TEST USED**	**STATISTIC**	**P VALUE**
S3A	IgG=6, anti-CD122=5<br>for each panel	Normal distribution	Unpaired t test	t(TRL)=2.568;<br>t(CD3 T)=1.637;<br>t(CD19⁺ B)=0.9097;<br>t(NK)=2.406;<br>t(neutrophil)=2.566;<br>t(DC)=2.203;<br>t(monocyte)=1.124	p(TRL)=0.0303;<br>p($CD3^+$ T)=0.1360;<br>p($CD19^+$ B)=0.3867;<br>p(NK)=0.0395;<br>p(neutrophil)=0.0304;<br>p(DC)=0.0550;<br>p(monocyte)=0.2900
S3B	3d: IgG=7, anti-<br>CD25=6, anti-<br>CD122=7;<br>7d: IgG=5, anti-<br>CD25=8, anti-<br>CD122=5	Normal distribution	1-way ANOVA,<br>Dunnett post hoc	F(3d)=40.09;<br>F(7d)=61.23	p(3d)<0.0001, p(IgG vs anti-<br>CD25)>0.9999, p(IgG vs anti-<br>CD122)<0.0001, p(anti-CD25 vs anti-<br>CD122)<0.0001;<br>p(7d)<0.0001, p(IgG vs anti-<br>CD25)<0.0001, p(IgG vs anti-<br>CD122)<0.0001, p(anti-CD25 vs anti-<br>CD122)>0.9999
S3C<br>Adhesive	IgG sham=6;<br>Anti-CD122 sham=6	Normal distribution	2-way ANOVA,<br>Bonferroni post hoc	F(1,10)=0.7864	p=0.3960;<br>p>0.9999 for all time points<br>between two groups.
S3C<br>Rotarod	IgG sham=6;<br>Anti-CD122 sham=6	Normal distribution	2-way ANOVA,<br>Bonferroni post hoc	F(1,10)=0.0182	p=0.8953;<br>p>0.9999 for all time points<br>between two groups.
S3D	IgG=7, anti-<br>CD122+anti-CD25=6	Normal distribution	Unpaired t test	t=2.779	p=0.0179
S3F	3d: IgG=8, anti-NK<br>1.1=6;<br>7d: IgG=8, anti-<br>NK1.1=8	Normal distribution	Student's t test	3d t=1.882<br>7d t=1.094	p(3d)=0.082;<br>p(7d)=0.2924
**FIGURE S5**	**n**	**DATA STRUCTURE**	**TEST USED**	**STATISTIC**	**P VALUE**
S5 C	d0: blood=3,<br>spleen=3, ipsilateral<br>brain=4;<br>d1: blood=7,<br>spleen=6, ipsilateral<br>brain=8;<br>d3: blood=7,<br>spleen=6, ipsilateral<br>brain=5;<br>d5: blood=5,<br>spleen=5, ipsilateral<br>brain=5;	Non normal<br>distribution	1-way ANOVA,<br>Bonferroni post hoc<br>for each timepoint	1d: F(2,18)=6.223,<br>3d: F(2,15)=6.254,<br>5d: F(2,12)=18.59	1d:<br>p=0.0088,<br>p(blood vs spleen)=0.4105,<br>p(blood vs ipsilateral brain)=0.1911,<br>p(spleen vs ipsilateral<br>brain)=0.0077;<br>3d:<br>p=0.0106,<br>p(blood vs spleen)>0.9999<br>p(blood vs ipsilateral brain)=0.0555<br>p(spleen vs ipsilateral<br>brain)=0.0111;<br>5d:<br>p=0.0002,<br>p(blood vs spleen)>0.9999,<br>p(blood vs ipsilateral brain)=0.0007<br>p(spleen vs ipsilateral brain)=0.0005
S5D	Blood=3, spleen=3,<br>ipsilateral brain=5	Normal distribution	1-way ANOVA,<br>Bonferroni post hoc	F (2,8)=82.96	p<0.0001;<br>p(blood vs spleen)>0.9999;<br>p(blood vs ipsilateral brain)<0.0001;<br>p(spleen vs ipsilateral brain)<0.0001
S5 F	Anti-CD122=6, WT<br>TRL=8, KO TRL=8	Normal distribution	1-way ANOVA,<br>Bonferroni post hoc	F (2,19)=15.89	p<0.0001;<br>p(anti-CD122 vs WT TRL)<0.0001,<br>p(anti-CD122 vs KO TRL)=0.0948,<br>p(WT TRL vs KO TRL)=0.0075
S5 G	n=4 for both WT TRL<br>and KO TRL	Normal distribution	Unpaired t test	t(CCL1)=6.453;<br>t(CCL2)=4.549;	p(CCL1)=0.0007;<br>p(CCL2)=0.0039;

	n	DATA STRUCTURE	TEST USED	STATISTIC	P VALUE
				t(CCL4)=2.380; t(CCL5)=2.286; t(IL6)=3.195; t(IL17A)=2.170; t(IFNg)=0.4778; t(MMP9)=0.0276.	p(CCL4)=0.0548; p(CCL5)=0.0623; p(il6)=0.0187; p(IL17A)=0.0730; p(IFNg)=0.6497 p(MMP9)=0.9788
**FIGURE S6**	**n**	**DATA STRUCTURE**	**TEST USED**	**STATISTIC**	**P VALUE**
S6A wb erk	*N=5*	*Normal distribution*	*1-way ANOVA, Bonferroni post hoc*	*F (9,40)=53.53*	*p<0.0001; p(ctrl vs ETGF)=0.0012, p(ctrl vs OGD 0.5h)<0.0001, p(ctrl vs OGD 0.5h ETGF)<0.0001, p(ctrl vs OGD 2h)<0.0001, p(ctrl vs OGD 2h ETGF)<0.0001; p(OGD 6h vs OGD 6h ETGF)=0.0295, p(OGD 24h vs OGD 24h ETGF)<0.0001*
S6A wb Akt	*N=5*	*Normal distribution*	*1-way ANOVA, Bonferroni post hoc*	*F (9,40)=13.39*	*p<0.0001; p(ctrl vs OGD 0.5h ETGF)<0.0001, p(OGD 24h vs OGD 24h ETGF)=0.024*
S6B	*N=4*	*Normal distribution*	*1-way ANOVA, Bonferroni post hoc*	*F (4,15)=15.67*	*p<0.0001; p(ctrl vs OGD)<0.0001, p(OGD vs OGD ETGF)=0.0068, p(OGD ETGF vs OGD ETGF U)=0.0183, p(OGD ETGF vs OGD ETGF A)=0.0163*
S6C	*N=4*	*Normal distribution*	*1-way ANOVA, Bonferroni post hoc*	*F (4,15)=69.16*	*p<0.0001; p(ctrl vs OGD)<0.0001, p(OGD vs OGD ETGF)<0.0001, p(OGD ETGF vs OGD ETGF U)<0.0001, p(OGD ETGF vs OGD ETGF A)<0.0001*
**FIGURE S7**	**n**	**DATA STRUCTURE**	**TEST USED**	**STATISTIC**	**P VALUE**
S7C	*PBS=6, Treg=8*	*Normal distribution*	*Unpaired t test*	*t=2.273*	*P=0.0422*
S7D	*Sham young=6, sham aged=5, young 3=6,aged 3=7, young 5=6, aged=6*	*Normal distribution*	*Sham: Welch's t test; 3d: unpaired t test; 5d: Welch's t test*	*Sham: Welch-corrected t=2.504; 3d: t=2.701; 5d: Welch-corrected t=1.159*	*p(sham)=0.0597, p(3d)=0.0206, p(5d)=0.2771*
S7F	*PBS=10; TRLs=7*	*Normal distribution*	*2-way ANOVA, Bonferroni post hoc*	*F(1,15)=57.71*	*p<0.0001; p(Pre)>0.9999; p(3d)<0.0001; p(7d)<0.0001; p(14d)=0.0009; p(21d)=0.0004; p(28d)=0.0074; p(35d)>0.9999*
S7G	*PBS=10; TRLs=7*	*Normal distribution*	*2-way ANOVA, Bonferroni post hoc*	*F(1,15)=71.72*	*p<0.0001; p(Pre)>0.9999; p(3d)<0.0001; p(7d)<0.0001; p(14d)<0.0001; p(21d)=0.0105; p(28d)=0.0075; p(35d)=0.0321*
S7H	*PBS=11; TRLs=8*	*Normal distribution*	*2-way ANOVA, Bonferroni post hoc*	*F(1,17)=19.08*	*P=0.0004; p(21d)=0.5889; p(22d)<0.0001; p(23d)=0.0052; p(24d)=0.1114*
S7I	*PBS=11; TRLs=8*	*Normal distribution*	*Unpaired t test*	*t=3.981*	*p=0.0010*
S7J	*PBS=10; TRLs=7*	*Normal distribution*	*Unpaired t test*	*t=6.882*	*p<0.0001*
S7K	*PBS=7; TRLs=7*	*Normal distribution*	*Unpaired t test*	*t=3.355*	*p=0.0057*
